# Supplementary material for: Integration of Multi-Omics, Histological, and Biochemical Analysis Reveals the Toxic Responses of Nile Tilapia Liver to Chronic Microcystin-LR Exposure
Source: Toxins (Basel). 2024 Mar 14;16(3):149. doi: 10.3390/toxins16030149 (PMC10974751; doi:10.3390/toxins16030149)
Supplement: Supplementary file 1 [file toxins-16-00149-s001.zip › toxins-2873506-supplementary.pdf]

Supplementary Materials: Integration of Multi-Omics, Histological, and Biochemical Analysis Reveals the Toxic Responses of Nile Tilapia Liver to Chronic Microcystin-LR Exposure

Yichao Li, Huici Yang, Bing Fu, Gen Kaneko, Hongyan Li, Jingjing Tian, Guangjun Wang, Mingken Wei, Jun Xie and Ermeng Yu

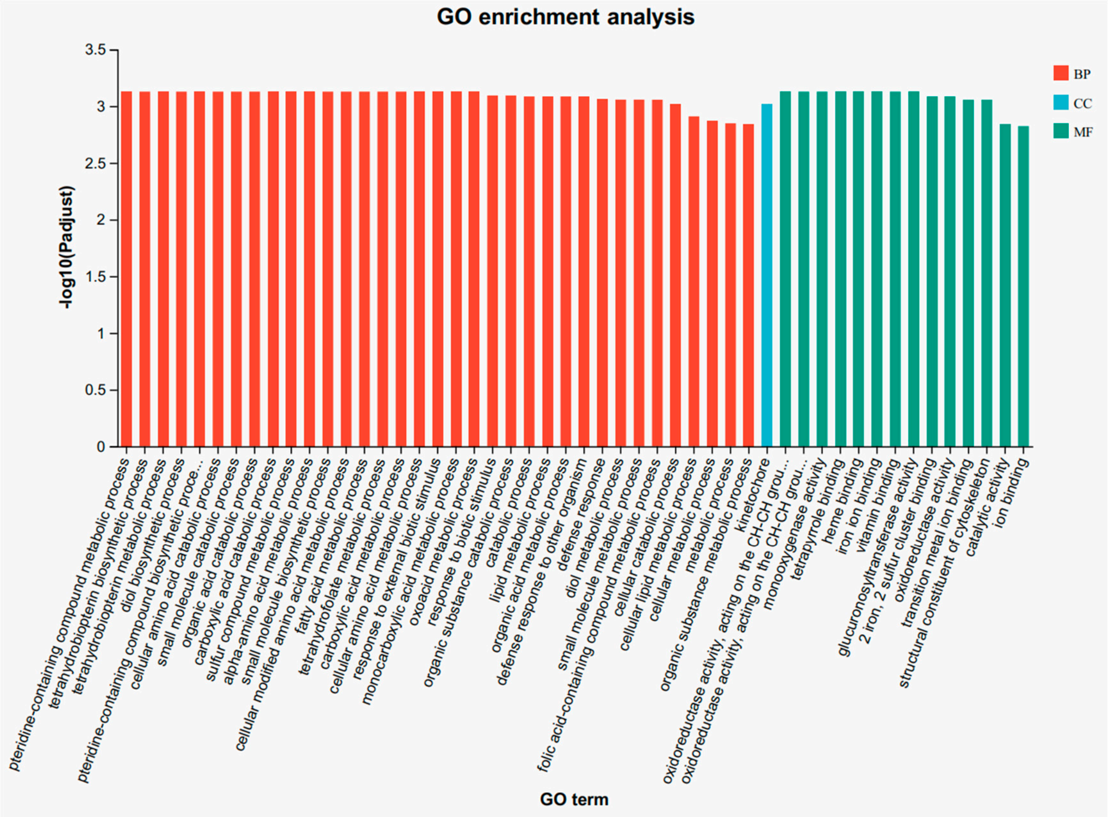

Figure S1. GO enrichment analysis of differential expressed genes.

Table S1. The composition of experimental diet ingredients (g/kg).

Ingredients	g/kg	Nutrients content	(%)
soybean meal	300	crude protein	30.79
cottonseed meal	120	crude fat	5.97
rapeseed meal	200	moisture	10.03
wheat flour	120		
rice bran	108		
wheat bran	80		
Soybean oil	30		
choline chloride	2		
mineral mixture	20		
vitamin mixture	20		

Note: Mineral mixture: iron 5 g/kg, copper 100 mg/kg, zinc 1.5 g/kg, manganese 0.5 g/kg, magnesium 20 g/kg, iodine 15 mg/kg, cobalt 5 mg/kg, selenium 5 mg/kg, and sodium, calcium, etc. Vitamin mixture: vitamin A 180000 IU/kg, vitamin D3 40,000 IU/ kg, vitamin E 1.6 g/kg、vitamin K3 0.0.5 g/kg, vitamin B1 0.2 g/kg, vitamin B2 0.25 g/kg, vitamin B6 0.2 g/kg, vitamin B12 0.65 mg/kg, vitamin C 2.5 g/kg, niacin 0.65 g/kg, calcium pantothenate 0.65 g/kg, folic acid 0.03 g/kg, Inositol 1 g/kg, Biotin H 8.3 mg/kg.

Table S2. Primers used for the analysis of mRNA genes expression by qRT-PCR.

Primes	Gene name	Sequences	NCBI number/Reference
cyp7a1	cholesterol 7-alpha-monooxygenase	F: GGGATAAGACACAGGCAACCA<br>R: TGCGGAGGAATTGAAGTGGG	XM_003456729.5
cpt1a	carnitine o-palmitoyltransferase 1	F: TTTCCAGGCCTCCTTACCCA<br>R: TTGTACTGCTCATTGTCCAGCAGA	XM_003440552
acox3	acyl-coenzyme a oxidase 3	F: AGGAGTCTCAGGAGATCCTCAAA<br>R: CCAGTGACCTGCAGTAGTAAACT	XM_013266255
acaa2	acetyl-CoA acyltransferase 2	F: AACGCTTCAGGTGTATCT<br>R: AAGTGCTTCTGTGATTGC	[50]
hadhb	trifunctional multienzyme complex subunit beta	F: CATGGCAAATCTGAAGGCTATGG<br>R: CAGTCTGTTTGCTACTGTGGTTG	XM_003446255.4
acadm	acyl-CoA dehydrogenase medium chain	F: CCCTTGGCTTCTCATTTGAGTTG<br>R: ACCACTCTTGTCATAAGCAGGAG	XM_003448445.4
sod	superoxide dismutase	F: GACGTGACAACACAGGTTGC<br>R: TACAGCCACCGTAACAGCAG	XM_003449940.5
cat	catalase	F: TCAGCACAGAAGACACAGACA<br>R: GACCATTCCTCCACTCCAGAT	XM_031754288.1
gst	glutathione S-transferases	F: TAATGGGAGAGGGAAGATGG<br>R: CTCTGCGATGTAATTCAGG	NM_001279635.1
g6pd	glucose-6-phosphate dehydrogenase	F: GAGAAGCCCTTTGGTCGTGA<br>R: ATCAAAGTACCCTCCACGGC	XM_005478106
caspase-3	cysteine-aspartic acid protease 3	F: GGAGTGGACGATACAGACGCAAA<br>R: TGAAGCTGTGTGACTGGGGCTT	NM_001282894.1
bcl2	B-cell lymphoma-2	F: GACGATGATGCCAGGGAGAG<br>R: CTCAGAGTTCACTGGAGCGG	XM_003437902.5
xiap	X-linked inhibitor of apoptosis protein	F: TCCAGACAGGTGTAGAGGAGG<br>F: CGCACTGGAAACACAAGACTG	XM_003445817.5
atg4b	autophagy related 4B cysteine peptidase	F: CCCGATGAGACGTACCACTG<br>R: CCATATGAGAGGGCTGGCTG	XM_003444208.4
atg5	autophagy related 5	F: ACAGCGTCTTACCCTGGAGCA<br>R: TCACAGAGCTGGATGGGCAGT	XM_003450274.5
atg12	autophagy related 12	F: ACAGTACAGTCACTCGCTCA<br>R: AAAACACTCGAAAAGCACACC	XM_019125508.1
β-actin	β-actin	F: AGCCTTCCTTCCTTGGTATGGAAT<br>R: TGTTGGCGTACAGGTCCTTACG	KJ126772

50. Yu, L.; Tian, J.; Zhang, C.; Xue, Z.; Lu, X.; Wen, H.; Jiang, M.; Wu, F. Acetylferulic Paeonol Ester: A New Feed Additive Reduces Lipid Accumulation in the Liver of Nile Tilapia (Oreochromis Niloticus) by Modulating Lipid and Glucose Metabolism. *Aquaculture* **2022**, *561*, 738671

Table S3. Differential metabolites in the Nile tilapia liver of the M30 group and control group (C) (n = 6).

	ID	Metabolite	Metab ID	CAS ID	M/Z	Retention time	Adducts	Formula	VIP	FC (M30/C)	P_value
1	neg_6394	(6R)-Folinic acid	metab_13365	-	472.16	2.92	M-H	C20H23N7O7	2.25	0.35	0.01
2	pos_3149	14-Hydroperoxy-H4-neuroprostane	metab_2335	-	415.21	4.26	M+Na	C22H32O6	1.70	0.50	0.00
3	pos_4663	N,N,O-Tridesmethylvenlafaxine	metab_3967	149289-29-2	218.15	5.63	M+H-H2O	C14H21NO2	1.79	0.61	0.00
4	neg_2386	Albifylline	metab_9073	-	261.14	3.14	M-H2O-H	C13H20N4O3	1.86	0.64	0.00
5	pos_5410	(+/-)-Hexanoylcarnitine	metab_4789	-	260.19	3.67	M+H	C13H25NO4	1.61	0.66	0.00
6	pos_6867	3-Methylguanine	metab_6362	2958-98-7	394.15	1.55	2M+ACN+Na	C6H7N5O	1.72	0.68	0.00
7	pos_4406	Alpha-Codeimethine	metab_3688	-	331.20	5.91	M+NH4	C19H23NO3	1.45	0.68	0.01
8	pos_3500	Cannabidiol	metab_2725	13956-29-1	353.19	5.62	M+K	C21H30O2	1.48	0.70	0.00
9	neg_2307	10-Formyldihydrofolate	metab_8990	28459-40-7	470.14	3.00	M-H	C20H21N7O7	1.75	0.70	0.00
10	neg_2792	Loganic Acid	metab_9502	22255-40-9	411.11	3.86	M+Cl	C16H24O10	1.60	0.71	0.00
11	neg_318	Oxidized glutathione	metab_9922	27025-41-8;15718-51-1	611.15	1.99	M-H, M+Na-2H	C20H32N6O12S2	1.18	0.71	0.00
12	pos_6999	PI-103	metab_6505	-	371.11	1.15	M+Na	C19H16N4O3	1.59	0.72	0.00
13	pos_3726	Docosadienoate (22:2n6)	metab_2964	17735-98-7	354.34	6.29	M+NH4	C22H40O2	1.45	0.72	0.00
14	pos_6760	6-(4-methyl-2-oxopentyl)-4-hydroxy-2-pyrone	metab_6252	-	242.11	1.72	M+CH3OH+H	C11H13O4-	1.49	0.72	0.00
15	pos_5335	L-Hexanoylcarnitine	metab_4706	22671-29-0	260.19	3.83	M+H	C13H25NO4	1.53	0.72	0.00
16	pos_3506	Ritalinic acid	metab_2731	19395-41-6	202.12	5.63	M+H-H2O	C13H17NO2	1.27	0.73	0.00

17	neg_1602	(1R)-Hydroxy-(2R)-glutathionyl-1,2-dihydronaphthalene	metab_8273	-	450.14	1.71	M-H	C20H25N3O7S	1.68	0.73	0.00
18	pos_921	Ethoxyquin	metab_7561	91-53-2	218.15	6.12	M+H, 2M+H	C14H19NO	1.32	0.74	0.01
19	pos_504	Polymyxin B2	metab_4381	-	617.36	2.32	M+2Na+H, M+2Na	C55H96N16O13	1.42	0.75	0.01
20	neg_6965	2-(N-Morpholino)-ethanesulfonic acid	metab_13950	-	194.05	2.10	M-H	C6H13NO4S	1.52	0.75	0.00
21	pos_855	7-Hydroxyetodolac	metab_7487	-	304.16	5.57	M+H, M+Na	C17H21NO4	1.46	0.75	0.00
22	neg_7463	3-Hydroxynevirapine glucuronide	metab_14443	-	975.31	1.10	2M+Hac-H	C21H22N4O8	1.43	0.76	0.04
23	pos_2054	(5S,6R)-Methyl 5,6,7-trihydroxyheptanoate	metab_1141	-	234.13	1.81	M+ACN+H	C8H16O5	1.34	0.77	0.00
24	neg_3647	Cholic Acid	metab_10425	81-25-4	453.29	5.82	M+FA-H	C24H40O5	1.09	0.77	0.04
25	pos_5430	Isoketocamphoric acid	metab_4811	-	234.13	3.63	M+NH4	C10H16O5	1.32	0.77	0.00
26	neg_6496	(3S,4R,5R)-1,3,4,5,6-Pentahydroxy-1-morpholin-4-ylhexan-2-one	metab_13469	-	286.09	2.80	M+Na-2H	C10H19NO7	1.47	0.77	0.00
27	pos_6779	D-Xylono-1,5-lactone	metab_6268	-	314.11	1.68	2M+NH4	C5H8O5	1.40	0.77	0.00
28	pos_4276	N-(2-Hydroxyethyl)heptadecanamide	metab_3549	-	314.30	6.26	M+H	C19H39NO2	1.28	0.77	0.00
29	pos_2269	Glucaric acid	metab_1370	87-73-0	252.07	2.21	M+ACN+H	C6H10O8	1.18	0.77	0.00
30	pos_4813	Hydrocodone	metab_4131	125-29-1	317.19	5.29	M+NH4	C18H21NO3	1.20	0.78	0.01
31	pos_2018	7,8-Dihydrobiopterin	metab_1105	6779-87-9	240.11	1.69	M+H	C9H13N5O3	1.23	0.78	0.01
32	pos_4721	L-Octanoylcarnitine	metab_4031	25243-95-2	288.22	5.54	M+H	C15H29NO4	1.33	0.78	0.00

33	pos_3514	Tanacetol B	metab_2740	86787-28-2	314.23	5.65	M+NH4	C17H28O4	1.29	0.79	0.00
34	pos_3863	Taurocholic acid	metab_3107	81-24-3	538.28	7.28	M+Na	C26H45NO7S	1.54	0.79	0.00
35	pos_3711	16-Nitroxystearate	metab_2948	-	350.30	6.17	M+H-2H2O	C22H43NO4	1.22	0.79	0.00
36	pos_2016	Coelenterazine	metab_1103	55779-48-1	446.15	1.68	M+Na	C26H21N3O3	1.40	0.79	0.00
37	pos_6142	Afzelechin 7-apioside	metab_5597	114590-44-2	470.14	2.48	M+ACN+Na	C20H22O9	1.32	0.79	0.00
38	neg_1688	2-Amino-7-[(2R)-2,3-dihydroxypropanoyl]-5,6,7,8-tetrahydro-3H-pteridin-4-one	metab_8345	-	276.07	1.92	M+Na-2H	C9H13N5O4	1.43	0.80	0.00
39	neg_1270	Taurine	metab_7955	107-35-7	249.02	0.84	2M-H	C2H7NO3S	1.25	0.80	0.04
40	neg_5766	N6-Methyl-2'-deoxyadenosine	metab_12704	2002-35-9	246.10	3.82	M-H2O-H	C11H15N5O3	1.24	0.80	0.01
41	neg_7710	Xi-2,3-Dihydro-3,5-dihydroxy-6-methyl-4H-pyran-4-one	metab_14677	-	203.06	0.79	M+FA-H	C7H10O4	1.29	0.81	0.01
42	pos_6780	N-Methyl-L-Proline	metab_6270	475-11-6	281.15	1.68	2M+Na	C6H11NO2	1.21	0.82	0.00
43	neg_3197	Lotaustralin	metab_9940	534-67-8	260.11	4.71	M-H	C11H19NO6	1.17	0.82	0.04
44	pos_1078	(10Z,12E)-Hexadecadienoylcarnitine	metab_88	-	396.31	5.88	M+H, M+2Na-H	C23H41NO4	1.32	0.82	0.00
45	pos_4588	3-Hydroxytetradecanoyl carnitine	metab_3883	282523-64-2	388.31	5.75	M+H	C21H41NO5	1.21	0.82	0.01
46	pos_5343	5-(4-Hydroxybenzyl)thiazolidine-2,4-dione	metab_4715	-	224.04	3.81	M+H	C10H9NO3S	1.04	0.82	0.02
47	neg_6998	O-Succinyl-L-homoserine	metab_13984	1492-23-5	218.07	2.08	M-H	C8H13NO6	1.32	0.82	0.00

48	neg_2376	Normorphine 3-glucuronide	metab_9062	-	468.13	3.12	M+Na-2H	C22H25NO9	1.39	0.82	0.00
49	neg_2433	Kojibiose	metab_9121	2140-29-6	377.09	3.18	M+Cl	C12H22O11	1.14	0.83	0.03
50	pos_1891	2-Amino-6-methyl-2,3,6,7-tetrahydro-1H-pteridin-4-one	metab_966	-	182.10	1.30	M+H	C7H11N5O	1.14	0.83	0.00
51	pos_4628	Algestone	metab_3928	595-77-7	364.25	5.69	M+NH4	C21H30O4	1.10	0.83	0.00
52	neg_2278	S-Glutaryldihydrolipoamide	metab_8960	-	366.11	2.95	M+FA-H	C13H23NO4S2	1.34	0.83	0.00
53	pos_584	4-[(2,4-Dihydroxy-3,3-dimethylbutanoyl)amino]butanoic acid	metab_5263	-	234.13	2.91	M+H, M+Na	C10H19NO5	1.28	0.83	0.00
54	pos_4593	Hexadecanedioic acid mono-L-carnitine ester	metab_3889	42150-38-9	412.31	5.74	M+H-H2O	C23H43NO6	1.14	0.83	0.01
55	pos_1068	2-Hydroxymyristoylcarnitine	metab_77	-	370.29	5.89	M+H-H2O, M+H	C21H41NO5	1.26	0.83	0.00
56	neg_6681	Omega-Salicoyisalicin	metab_13662	-	441.09	2.53	M+Cl	C20H22O9	1.37	0.84	0.00
57	neg_7471	Gamma-Glutamyl-beta-cyanoalanine	metab_14451	16051-95-9	288.08	1.09	M+FA-H	C9H13N3O5	1.26	0.84	0.00
58	neg_2445	Lamivudine sulfoxide	metab_9132	-	266.02	3.20	M+Na-2H	C8H11N3O4S	1.20	0.84	0.00
59	pos_6522	Dipropyl disulfide	metab_6001	629-19-6	151.06	2.02	M+H	C6H14S2	1.17	0.84	0.00
60	neg_927	20-Cooh ltb4	metab_15324	80434-82-8	425.22	4.03	M+Cl, M+FA-H	C21H32O6	1.23	0.84	0.01
61	neg_6943	Neopterin	metab_13927	2277-42-1;2009-64-5;10162-32-0	565.18	2.10	2M+Hac-H	C9H11N5O4	1.31	0.84	0.00

62	pos_7130	N-formimidoyl-glutamic acid	metab_6644	816-90-0	175.07	0.95	M+H	C6H10N2O4	1.23	0.84	0.00
63	pos_6725	L-Histidinol	metab_6215	4836-52-6	174.12	1.83	M+CH3OH+H	C6H11N3O	1.16	0.84	0.00
64	pos_4615	Lauroylcarnitine	metab_3914	25518-54-1	366.26	5.71	M+Na	C19H37NO4	1.13	0.85	0.00
65	neg_2610	1,2-Epoxy-3-(p-nitrophenoxy)propane	metab_9306	5255-75-4	194.05	3.52	M-H	C9H9NO4	1.22	0.85	0.00
66	pos_4255	PC(18:4(6Z,9Z,12Z,15Z)/18:3(6Z,9Z,12Z))	metab_3529	-	776.52	6.36	M+H	C44H74NO8P	1.10	0.85	0.01
67	neg_1365	3,5,6-Trihydroxy-5-(hydroxymethyl)-2-methoxy-2-cyclohexen-1-one	metab_8042	76663-30-4	203.06	0.99	M-H	C8H12O6	1.22	0.85	0.00
68	pos_410	Ketone Ester	metab_3366	-	218.14	1.91	M+NH4, M+ACN+H	C8H16O4	1.20	0.85	0.00
69	neg_7428	D-Arabinono-1,4-lactone	metab_14407	322392.00	147.03	1.17	M-H	C5H8O5	1.28	0.85	0.02
70	pos_2042	8-Hydroxyguanosine	metab_1128	3868-31-3	322.08	1.75	M+Na	C10H13N5O6	1.09	0.86	0.00
71	neg_1447	Cytosine deoxyribonucleoside	metab_8121	951-77-9	272.09	1.18	M+FA-H	C9H13N3O4	1.03	0.86	0.02
72	pos_6886	SCHEMBL13960539	metab_6383	-	501.19	1.50	M+K	C26H30N4O4	1.15	0.86	0.00
73	pos_4629	Decanoylcarnitine	metab_3929	1492-27-9	316.25	5.69	M+H	C17H33NO4	1.07	0.86	0.00
74	neg_4920	Y1 Receptor antagonist 1	metab_11781	-	486.25	5.59	M-H	C28H33N5O3	1.05	0.86	0.02
75	pos_7101	Succinic acid semialdehyde	metab_6613	692-29-5	205.07	0.99	2M+H	C4H6O3	1.10	0.86	0.00

76	pos_4552	6-Hydroxyhexadecanoylcarnitine	metab_3845	-	398.33	5.78	M+H-H2O	C23H45NO5	1.12	0.87	0.00
77	neg_7866	5-L-Glutamyl-taurine	metab_14832	56488-60-9	253.05	0.63	M-H	C7H14N2O6S	1.21	0.87	0.00
78	pos_6715	Galactaric acid	metab_7660	526-99-8	252.07	1.83	M+ACN+H	C6H10O8	1.06	0.87	0.00
79	pos_4266	Paullinic acid	metab_3541	17735-94-3	328.32	6.32	M+NH4	C20H38O2	1.09	0.87	0.00
80	neg_5199	Tranexamic Acid	metab_12087	1197-18-8	202.11	4.87	M+FA-H	C8H15NO2	1.05	0.87	0.00
81	pos_899	Fluocortin butyl	metab_7535	-	488.28	5.90	M+NH4, M+ACN+H	C26H35FO5	1.14	0.87	0.00
82	pos_1275	N-Acetylasparagine	metab_306	4033-40-3	175.07	0.66	M+H-H2O, M+H	C6H10N2O4	1.17	0.87	0.00
83	pos_1115	MG(20:5(5Z,8Z,11Z,14Z,17Z)/0:0/0:0)	metab_129	-	394.29	5.75	M+NH4, M+ACN+H	C23H36O4	1.04	0.87	0.00
84	neg_2114	Formycin b	metab_8784	-	249.06	2.70	M-H2O-H	C10H12N4O5	1.20	0.88	0.00
85	pos_3743	PC(18:4(6Z,9Z,12Z,15Z)/14:0)	metab_2983	-	726.51	6.39	M+H	C40H72NO8P	1.06	0.88	0.00
86	pos_3759	PC(14:0/18:3(9Z,12Z,15Z))	metab_2999	-	728.52	6.48	M+H	C40H74NO8P	1.02	0.88	0.01
87	pos_1558	L-Carnitine	metab_614	541-15-1	162.11	0.61	M+H	C7H15NO3	1.17	0.88	0.00
88	neg_2134	8-Oxo-dGMP	metab_8805	127027-50-3	384.03	2.73	M+Na-2H	C10H14N5O8P	1.08	0.88	0.00
89	pos_6304	Lysocellin	metab_5772	-	337.20	2.25	M+2Na	C34H60O10	1.13	0.88	0.00
90	pos_301	Cytidine	metab_2181	65-46-3	244.09	1.02	M+H, 2M+H	C9H13N3O5	1.15	0.88	0.00
91	pos_1260	Citrulline	metab_290	372-75-8	176.10	0.62	M+H, M+K	C6H13N3O3	1.00	0.88	0.00
92	pos_6481	Zeranol	metab_7676	36455-72-8;26538-44-3	367.15	2.03	M+2Na-H	C18H26O5	1.02	0.89	0.00
93	pos_7354	Cytarabine	metab_6878	147-94-4	244.09	0.69	M+H	C9H13N3O5	1.04	0.89	0.00

94	neg_1930	4-(4-Fluorophenyl)-3-(4-hydroxy-3-methoxyphenoxymethyl)piperidine	metab_8595	-	352.13	2.38	M+Na-2H	C19H22FNO3	1.01	0.89	0.00
95	neg_7593	Toralactone	metab_14566	41743-74-2	253.05	0.90	M-H2O-H	C15H12O5	1.09	0.89	0.00
96	neg_7209	Citramalic Acid	metab_14202	597-44-4;2306-22-1	147.03	1.78	M-H	C5H8O5	1.06	0.89	0.01
97	pos_6599	Trans-3,4-Dihydro-3,4-dihydroxy-7,12-dimethylbenz[a]anthracene	metab_6083	68162-13-0	354.15	1.94	M+ACN+Na	C20H18O2	1.02	0.90	0.00
98	pos_6740	Morph	metab_6230	57-27-2	261.14	1.78	M+CH3OH+H	C10H16N2O4	1.04	0.90	0.00
99	neg_1865	Astringin	metab_8531	29884-49-9	441.09	2.13	M+Cl	C20H22O9	1.11	0.90	0.00
100	neg_7235	LEUCOGENENOL	metab_14220	-	404.13	1.71	M+Na-2H	C18H25NO8	1.01	0.90	0.00
101	neg_7082	4-Aminophenylmannoside	metab_14074	-	306.08	2.00	M+Cl	C12H17NO6	1.01	0.90	0.00
102	neg_7611	3-Phosphoglycerate	metab_15402	820-11-1;3443-58-1	184.99	0.88	M-H	C3H7O7P	1.02	0.90	0.00
103	neg_2244	Quercetin 3-gluco-xyloside	metab_8923	-	577.12	2.90	M-H2O-H	C26H28O16	1.06	0.91	0.00
104	neg_6997	Lysozyme	metab_13983	-	250.06	2.08	M+FA-H	C8H15NOS2	1.02	0.91	0.00
105	neg_4615	Oxymesterone	metab_11456	145-12-0	317.21	6.01	M-H	C20H30O3	1.01	0.91	0.00
106	neg_2491	FAD	metab_9177	146-14-5	784.15	3.25	M-H	C27H33N9O15P2	1.01	0.92	0.00

107	pos_345	Isobutylidene	metab_2668	-	175.12	0.57	M+H-H2O, M+H	C6H14N4O2	1.03	1.10	0.00
108	neg_5034	Stercobilin	metab_11906	34217-90-8	629.32	5.35	M+Cl	C33H46N4O6	1.02	1.11	0.00
109	pos_54	V-PYRRO/NO	metab_4776	-	175.12	0.81	M+H, M+NH4, M+H-2H2O	C6H11N3O2	1.04	1.11	0.00
110	neg_7503	3-Methylthiopropionic acid	metab_14482	646-01-5	165.02	1.03	M+FA-H	C4H8O2S	1.11	1.12	0.00
111	pos_2312	Gly-Ile	metab_1417	-	189.12	2.28	M+H	C8H16N2O3	1.00	1.12	0.02
112	neg_656	Fe(II)-nicotianamine	metab_13537	-	302.14	2.47	M-H, M+Na-2H	C12H21N3O6	1.09	1.12	0.01
113	neg_2494	Leucyl-Methionine	metab_9180	-	261.13	3.27	M-H	C11H22N2O3S	1.01	1.13	0.03
114	neg_6774	Eltoprazine	metab_13756	-	265.12	2.41	M+FA-H	C12H16N2O2	1.02	1.13	0.03
115	pos_2010	L-Theanine	metab_1097	3081-61-6	175.11	1.65	M+H	C7H14N2O3	1.00	1.13	0.02
116	pos_6967	Prostratin	metab_6472	60857-08-1	435.17	1.21	M+2Na-H	C22H30O6	1.04	1.13	0.00
117	neg_7971	2-Amino-4-[carbamimidoyl(methyl)amino]butanoic acid	metab_14935	-	173.10	0.56	M-H	C6H14N4O2	1.08	1.13	0.01
118	neg_6543	Undeca-4,6,8-trienedioylcarnitine	metab_13520	-	374.16	2.74	M+Na-2H	C18H27NO6	1.06	1.13	0.01
119	pos_2463	Deoxypyridinoline	metab_1579	83462-55-9	445.23	2.56	M+CH3OH+H	C18H28N4O7	1.03	1.13	0.01
120	neg_6614	6-Hydroxymelatonin	metab_13595	2208-41-5	293.11	2.62	M+FA-H	C13H16N2O3	1.06	1.13	0.03

121	pos_6245	4-Hydroxyproline	metab_7649	51-35-4;618-27-9	304.15	2.32	2M+ACN+H	C5H9NO3	1.02	1.13	0.00
122	neg_340	5-(Hydroxymethyl)-1H-pyrrole-2-carbaldehyde	metab_10165	-	309.11	2.08	2M-H, 2M+Hac-H	C6H7NO2	1.08	1.13	0.01
123	pos_369	Gln Val	metab_2925	-	246.14	1.64	M+H-H2O, M+H	C10H19N3O4	1.02	1.13	0.01
124	neg_8121	Seryllysine	metab_15099	22677-61-8	232.13	0.50	M-H	C9H19N3O4	1.03	1.13	0.02
125	pos_39	(2S)-2-Amino-6-[(3-formylpiperidin-1-yl)amino]hexanoic acid	metab_3145	-	275.21	0.48	M+2H, M+NH4, M+H	C12H23N3O3	1.04	1.13	0.00
126	pos_4222	1-O-Hexadecyl-2-C-Methyl-3-Phosphatidylcholine	metab_3494	-	496.38	6.53	M+H	C25H54NO6P	1.04	1.13	0.00
127	pos_5739	Isofebrifugine	metab_5151	32434-44-9	302.15	3.09	M+H	C16H19N3O3	1.00	1.13	0.02
128	neg_7825	Prolylglycine	metab_14794	2578-57-6	217.08	0.67	M+FA-H	C7H12N2O3	1.04	1.13	0.03
129	neg_1713	TETRAHYDROURIDINE	metab_8373	-	229.08	1.96	M-H2O-H	C9H16N2O6	1.04	1.13	0.03
130	neg_2175	ALA-ILE	metab_8848	29727-65-9	201.12	2.80	M-H	C9H18N2O3	1.10	1.13	0.03
131	neg_1968	Quinagolida	metab_8635	-	430.19	2.43	M+Cl	C20H33N3O3S	1.10	1.14	0.01
132	pos_1250	Pro Arg	metab_279	-	272.17	0.61	M+2H, M+H	C11H21N5O3	1.00	1.14	0.01
133	neg_660	H-Tyr-gly-gly-OH	metab_13580	-	294.11	2.48	M-H, M+FA-H	C13H17N3O5	1.04	1.14	0.02
134	neg_7191	Methionyl-Glycine	metab_14184	-	205.06	1.82	M-H	C7H14N2O3S	1.10	1.14	0.02
135	pos_1224	Histidylleucine	metab_250	7763-65-7	269.16	1.49	M+H-H2O, M+H	C12H20N4O3	1.10	1.14	0.01

136	pos_6778	Ile Thr	metab_6267	-	233.15	1.69	M+H	C10H20N2O4	1.02	1.14	0.01
137	neg_808	Valylleucine	metab_15055	3989-97-7	229.16	3.25	M-H, 2M-H	C11H22N2O3	1.11	1.14	0.03
138	neg_2477	Phenol sulphate	metab_15433	937-34-8	172.99	3.24	M-H	C6H6O4S	1.21	1.14	0.00
139	neg_5654	Fasoracetam	metab_12583	110958-19-5	241.12	4.00	M+FA-H	C10H16N2O2	1.05	1.14	0.03
140	pos_1482	Ser Lys	metab_533	-	234.14	0.50	M+H	C9H19N3O4	1.02	1.14	0.01
141	neg_6359	Neuromedin B (1-3)	metab_13326	-	301.15	2.97	M-H	C12H22N4O5	1.10	1.14	0.01
142	pos_7684	Alanyllysine	metab_7237	6366-77-4	218.15	0.50	M+H	C9H19N3O3	1.05	1.14	0.01
143	pos_5136	Ser Ile Phe	metab_4487	-	366.20	4.28	M+H	C18H27N3O5	1.01	1.14	0.00
144	pos_1124	Ile Thr Phe	metab_139	-	380.22	4.38	M+H, M+Na	C19H29N3O5	1.03	1.14	0.00
145	pos_5017	Ser Val Gln Leu Leu	metab_4357	-	559.34	4.54	M+H	C25H46N6O8	1.03	1.14	0.00
146	pos_6879	N(epsilon)-(carboxyethyl)lysine	metab_6375	-	219.13	1.52	M+H	C9H18N2O4	1.09	1.14	0.00
147	neg_5937	PS(5-iso PGF2VI/14:1(9Z))	metab_12890	-	812.38	3.54	M+K-2H	C38H66NO13P	1.03	1.14	0.04
148	neg_2747	Leucylleucine	metab_9453	3303-31-9	243.17	3.78	M-H	C12H24N2O3	1.07	1.14	0.05
149	pos_384	N6-Acetyl-L-lysine	metab_3082	692-04-6	189.12	1.60	M+H, 2M+H	C8H16N2O3	1.06	1.14	0.01
150	pos_1608	Valyllysine	metab_665	22677-62-9	246.18	0.69	M+H	C11H23N3O3	1.12	1.14	0.00
151	neg_7857	Threonylalanine	metab_14824	56217-50-6	189.09	0.64	M-H	C7H14N2O4	1.05	1.14	0.01
152	neg_4990	Phe-Pro-Ile	metab_11857	-	420.21	5.50	M+FA-H	C20H29N3O4	1.08	1.14	0.00
153	pos_1373	Threoninyl-Gamma-glutamate	metab_415	-	248.12	0.92	M+H-H2O, M+H	C9H17N3O5	1.03	1.14	0.01

154	neg_7008	Ophthalmic acid	metab_13996	495-27-2	288.12	2.07	M-H	C11H19N3O6	1.12	1.14	0.00
155	pos_2728	Lys Leu Phe	metab_1869	-	407.26	3.13	M+H	C21H34N4O4	1.01	1.14	0.00
156	pos_1915	Val Thr His	metab_992	-	356.19	1.40	M+H	C15H25N5O5	1.09	1.14	0.00
157	neg_3754	Dehydroeburicoic acid	metab_10538	-	449.34	6.07	M-H2O-H	C31H48O3	1.08	1.14	0.00
158	pos_1148	Deoxycholylhistidine	metab_165	-	530.35	2.17	M+2H, M+H	C30H47N3O5	1.05	1.14	0.01
159	pos_6697	Tyr Glu	metab_6186	-	311.12	1.85	M+H	C14H18N2O6	1.02	1.14	0.01
160	neg_2020	Batatasin IV	metab_8692	60347-67-3	487.22	2.52	2M-H	C15H16O3	1.13	1.14	0.02
161	neg_2850	Isoleucyl-Leucine	metab_9566	-	243.17	3.97	M-H	C12H24N2O3	1.10	1.14	0.04
162	neg_4533	14-demethyllanosterol	metab_11369	2026401.00	455.35	6.13	M+FA-H	C29H46O	1.04	1.14	0.04
163	neg_1413	Tetrahydrothiophene-3-ol 1,1-dioxide	metab_8088	-	181.02	1.10	M+FA-H	C4H8O3S	1.09	1.14	0.00
164	neg_7993	Beta-Alanyl-L-lysine	metab_14958	-	216.14	0.56	M-H	C9H19N3O3	1.09	1.14	0.02
165	pos_2670	PIP(PGF2alpha/20:1(11Z))	metab_1805	-	522.28	3.01	M+2H	C49H88O19P2	1.06	1.14	0.01
166	pos_6545	Phenylalanylarginine	metab_6025	1238-09-1	322.19	1.99	M+H	C15H23N5O3	1.01	1.14	0.02
167	pos_6736	Thr Ile Lys Glu	metab_6226	-	490.29	1.80	M+H	C21H39N5O8	1.01	1.15	0.02
168	neg_2889	N-(3-acetamidopropyl)pyrrolidin-2-one	metab_9607	106692-36-8	229.12	4.05	M+FA-H	C9H16N2O2	1.01	1.15	0.01
169	neg_7200	Petasinine	metab_14195	70474-33-8	260.13	1.80	M+Na-2H	C13H21NO3	1.14	1.15	0.00
170	neg_857	Tyrosyl-Isoleucine	metab_15247	-	293.15	3.57	M-H, 2M-H	C15H22N2O4	1.16	1.15	0.02

171	neg_6741	Aminoglutethimide	metab_13721	125-84-8	277.12	2.47	M+FA-H	C13H16N2O2	1.06	1.15	0.03
172	pos_375	Ile Ser	metab_2990	-	260.16	1.70	M+H, M+ACN+H	C9H18N2O4	1.13	1.15	0.01
173	neg_6252	TRIETHYLENE GLYCOL	metab_13211	-	345.18	3.12	2M+FA-H	C6H14O4	1.22	1.15	0.01
174	pos_180	ALANYL-dl-LEUCINE	metab_869	-	203.14	2.26	M+H, M+Na, M+H-H2O	C9H18N2O3	1.09	1.15	0.02
175	pos_471	Gln Ile	metab_4018	-	260.16	2.21	M+H-H2O, M+H	C11H21N3O4	1.09	1.15	0.02
176	neg_5209	Tezampanel	metab_12099	-	557.33	4.85	2M-H	C13H21N5O2	1.18	1.15	0.00
177	pos_1898	Lys Val	metab_973	-	246.18	1.32	M+H	C11H23N3O3	1.10	1.15	0.01
178	neg_6529	Guanadrel	metab_13505	40580-59-4	258.15	2.76	M+FA-H	C10H19N3O2	1.17	1.15	0.02
179	pos_7489	(+)-gamma-Hydroxy-L-homoarginine	metab_7024	1616-99-5	246.16	0.58	M+ACN+H	C7H16N4O3	1.03	1.15	0.03
180	pos_6465	Glutamylphenylalanine	metab_5938	20556-22-3	606.27	2.03	2M+NH4	C14H18N2O5	1.01	1.15	0.02
181	neg_3013	L-Prolinamide,D-tyrosyl-L-arginylglycyl-4-nitro-L-phenylalanyl-	metab_9743	-	663.30	4.29	M-H2O-H	C31H42N10O8	1.16	1.15	0.00
182	pos_2779	Leu Glu Val	metab_1925	-	360.21	3.25	M+H	C16H29N3O6	1.09	1.15	0.00
183	neg_2295	Uridine, 2'-deoxy-2'-fluoro-2'-methyl-, (2'R)-	metab_8978	-	305.08	2.99	M+FA-H	C10H13FN2O5	1.21	1.15	0.00
184	neg_3258	4-(2,2,6,6-Tetramethyl-1-oxylpiperidin-4-ylamino)-4'-	metab_10008	-	535.24	4.87	M-H2O-H	C30H38N2O8	1.13	1.15	0.00

		demethylepipodophyllotoxin									
185	neg_1605	Ala Val Asp Asp	metab_8276	-	417.16	1.71	M-H	C16H26N4O9	1.12	1.15	0.03
186	pos_6859	Val Arg Ala	metab_6353	-	345.22	1.58	M+H	C14H28N6O4	1.09	1.15	0.00
187	pos_5246	Thr Ile Leu	metab_4607	-	346.23	4.02	M+H	C16H31N3O5	1.06	1.15	0.00
188	pos_5529	Sisomicin sulfate	metab_4918	-	430.27	3.43	M+H-H2O	C19H37N5O7	1.08	1.15	0.00
189	neg_223	Oxoglutaric acid	metab_8907	328-50-7	191.02	1.09	M-H2O-H, M+FA-H	C5H6O5	1.07	1.15	0.05
190	pos_1266	Thr Gln	metab_296	-	248.12	0.64	M+H-H2O, M+H	C9H17N3O5	1.07	1.15	0.01
191	pos_6457	2,3,4,5-Tetrahydro-2-pyridinecarboxylic acid	metab_5929	73980-78-6	272.16	2.04	2M+NH4	C6H9NO2	1.08	1.15	0.00
192	neg_7245	N2-Acetylornithine	metab_14228	1572591.00	173.09	1.70	M-H	C7H14N2O3	1.20	1.15	0.02
193	neg_61	Ser-Leu	metab_13067	6665-16-3	217.12	2.68	M-H, M+Cl, 2M-H	C9H18N2O4	1.20	1.15	0.03
194	neg_7520	N2-Succinyl-L-ornithine	metab_14501	899816-95-6	231.10	1.01	M-H	C9H16N2O5	1.07	1.15	0.04
195	pos_2833	Ile Glu Val	metab_1986	-	360.21	3.36	M+H	C16H29N3O6	1.12	1.15	0.00
196	neg_1971	Histidylarginine	metab_8639	77369-21-2	332.15	2.44	M+Na-2H	C12H21N7O3	1.22	1.15	0.01
197	pos_2516	Val Leu	metab_1637	-	231.17	2.67	M+H	C11H22N2O3	1.10	1.16	0.02
198	pos_6913	Tyr Lys	metab_6414	-	310.18	1.41	M+H	C15H23N3O4	1.12	1.16	0.00
199	pos_7515	D-Citrulline	metab_7053	-	158.09	0.57	M+H-H2O	C6H13N3O3	1.07	1.16	0.01
200	pos_2238	Ala Ile	metab_1339	-	203.14	2.12	M+H	C9H18N2O3	1.08	1.16	0.02
201	pos_5251	Ilicifolinoside A	metab_4613	-	546.28	4.00	2M+NH4	C11H20O7	1.10	1.16	0.00
202	neg_6755	Deca-2,5,8-trienedioylcarnitine	metab_13736	-	360.14	2.44	M+Na-2H	C17H25NO6	1.14	1.16	0.01

203	neg_1232	2-Amino-4-[(2-hydroxy-1-oxopropyl)amino]butanoic acid	metab_7916	38710-35-9	189.09	0.79	M-H	C7H14N2O4	1.05	1.16	0.02
204	pos_6573	Ecgonine Methyl Ester	metab_6056	1915211.00	217.15	1.95	M+NH4	C10H17NO3	1.07	1.16	0.02
205	neg_723	Threonylleucine	metab_14219	50299-12-2	231.13	2.84	M-H2O-H, M-H	C10H20N2O4	1.18	1.16	0.03
206	pos_465	Glycyl-leucine	metab_3952	869-19-2	189.12	2.17	M+H, 2M+H	C8H16N2O3	1.13	1.16	0.01
207	neg_2116	Asparaginylisoleucine	metab_8786	438533-59-6	244.13	2.71	M-H	C10H19N3O4	1.22	1.16	0.02
208	neg_3074	LF	metab_9806	424904.00	277.16	4.40	M-H	C15H22N2O3	1.18	1.16	0.04
209	neg_2325	Valylisoleucine	metab_9009	20556-14-3	229.16	3.03	M-H	C11H22N2O3	1.18	1.16	0.02
210	pos_480	Thr Ile	metab_4117	-	233.15	2.27	M+H, M+Na	C10H20N2O4	1.12	1.16	0.02
211	pos_412	Thr Glu Leu Lys	metab_3387	-	531.31	1.91	M+H, M+ACN+H	C21H39N5O8	1.14	1.16	0.01
212	pos_6868	Valylarginine	metab_6363	37682-75-0	274.19	1.55	M+H	C11H23N5O3	1.06	1.16	0.02
213	neg_6362	(S)-3-Hydroxy-N-methylcoclaurine	metab_13330	-	675.30	2.96	2M+FA-H	C18H21NO4	1.13	1.16	0.01
214	pos_2635	Agar	metab_1767	9002-18-0	319.14	2.93	M+H-H2O	C14H24O9	1.05	1.16	0.00
215	pos_5951	Methohexital	metab_5387	151-83-7	295.16	2.74	M+CH3OH+H	C14H18N2O3	1.03	1.16	0.03
216	neg_5829	N-Acetyl-leukotriene E4	metab_12771	80115-95-3	526.25	3.72	M+FA-H	C25H39NO6S	1.15	1.16	0.01
217	neg_3231	Phenylalanylisoleucine	metab_9979	22951-94-6	601.32	4.81	2M+FA-H	C15H22N2O3	1.22	1.16	0.00
218	neg_45	Gamma-Glu-Leu	metab_11331	2566-39-4	259.13	2.10	M-H, M+FA-H, M-H2O-H	C11H20N2O5	1.26	1.16	0.01
219	pos_1483	Asn Lys	metab_534	-	261.16	0.50	M+H	C10H20N4O4	1.09	1.16	0.01
220	neg_1564	N-alpha-Acetyl-L-lysine	metab_8237	1946-82-3	187.11	1.61	M-H	C8H16N2O3	1.25	1.16	0.01

221	neg_6108	Ptaquiloside	metab_13077	87625-62-5	379.17	3.26	M-H2O-H	C20H30O8	1.10	1.16	0.02
222	neg_3558	Apixaban	metab_10336	-	458.19	5.66	M-H	C25H25N5O4	1.12	1.16	0.00
223	pos_5407	Gamma-Glutamylisoleucine	metab_4785	23632-83-9	243.13	3.67	M+H-H2O	C11H20N2O5	1.12	1.16	0.01
224	pos_5631	Val Ile Val	metab_5032	-	330.24	3.26	M+H	C16H31N3O4	1.08	1.16	0.00
225	neg_7846	N-Acetyl-b-glucosaminylamine	metab_14816	-	219.10	0.66	M-H	C8H16N2O5	1.12	1.17	0.01
226	pos_5905	PA(PGF2alpha/19:2(10Z,13Z))	metab_5336	-	415.23	2.81	M+2Na	C42H73O11P	1.08	1.17	0.01
227	pos_1659	Gamma-glutamyl-L-putrescine	metab_718	-	218.15	0.80	M+H	C9H19N3O3	1.07	1.17	0.02
228	neg_8096	E-10-Hydroxydesmethylnortriptyline	metab_15072	-	300.12	0.51	M+Cl	C18H19NO	1.02	1.17	0.04
229	pos_4911	Leu Leu Leu	metab_4239	-	358.27	4.88	M+H	C18H35N3O4	1.08	1.17	0.00
230	pos_5748	Phe Met	metab_5161	-	297.13	3.08	M+H	C14H20N2O3S	1.09	1.17	0.00
231	pos_7108	Arg Val	metab_6619	-	274.19	0.99	M+H	C11H23N5O3	1.11	1.17	0.02
232	pos_1463	Lysyl-Lysine	metab_512	13184-13-9	138.11	0.48	M+2H	C12H26N4O3	1.10	1.17	0.00
233	neg_6270	CD 1790	metab_13230	-	494.19	3.09	M+Na-2H	C25H27N7O3	1.24	1.17	0.00
234	pos_3238	Ile Leu Ile	metab_2433	-	358.27	4.58	M+H	C18H35N3O4	1.08	1.17	0.00
235	pos_6783	Arg Leu	metab_6273	-	288.20	1.65	M+H	C12H25N5O3	1.24	1.17	0.00
236	pos_5020	Cyclo(Leu-Phe)	metab_4361	-	293.19	4.53	M+CH3OH+H	C15H20N2O2	1.03	1.17	0.00

237	pos_178	Ser Leu	metab_847	-	219.13	2.18	M+H, M+Na, M+H-H2O	C9H18N2O4	1.16	1.17	0.02
238	neg_7867	Methoxamine	metab_14833	390-28-3	232.09	0.63	M+Na-2H	C11H17NO3	1.19	1.17	0.01
239	neg_7036	Arginylleucine	metab_14026	1188-24-5	286.19	2.06	M-H	C12H25N5O3	1.18	1.17	0.02
240	neg_2523	L-Coprine	metab_9211	58919-61-2	403.18	3.32	2M-H	C8H14N2O4	1.27	1.17	0.01
241	pos_1965	Diaminopimelic acid	metab_1047	583-93-7	232.13	1.60	M+ACN+H	C7H14N2O4	1.15	1.17	0.00
242	pos_808	Ile Leu Phe	metab_7435	-	392.25	4.95	M+H, M+Na	C21H33N3O4	1.13	1.17	0.00
243	pos_597	Tyr Leu	metab_5407	-	295.16	2.99	M+H, M+Na	C15H22N2O4	1.19	1.17	0.01
244	pos_3176	Icariside B8	metab_2364	126176-78-1	421.24	4.34	M+CH3OH+H	C19H32O8	1.09	1.17	0.01
245	neg_1466	N2-Acetyl-L-ornithine	metab_8137	1572591.00	173.09	1.27	M-H	C7H14N2O3	1.15	1.17	0.04
246	neg_2714	Naftifine	metab_9418	65472-88-0	332.16	3.72	M+FA-H	C21H21N	1.14	1.17	0.00
247	pos_1265	Glutaminylalanine	metab_295	16874-70-7	218.11	0.62	M+H, M+K	C8H15N3O4	1.13	1.17	0.01
248	pos_7408	Meso-2,6-Diaminoheptanedioate	metab_7667	583-93-7	191.10	0.64	M+H	C7H14N2O4	1.06	1.17	0.02
249	neg_2597	Leucyl-leucine	metab_9292	2883-36-5;3303-31-9	243.17	3.49	M-H	C12H24N2O3	1.17	1.17	0.03
250	neg_7205	Acetylhomoserine	metab_14199	7540-67-2	381.15	1.78	2M+Hac-H	C6H11NO4	1.12	1.17	0.02
251	pos_548	Val Gln Val	metab_4864	-	345.21	2.65	M+H, M+Na	C15H28N4O5	1.12	1.17	0.01
252	neg_5306	Blumenol C O-[apiosyl-(1->6)-glucoside]	metab_12204	177261-68-6	539.23	4.63	M+Cl	C24H40O11	1.09	1.17	0.02
253	pos_2106	Ile Ala	metab_1197	-	203.14	1.90	M+H	C9H18N2O3	1.16	1.17	0.01
254	pos_5684	Statine	metab_5090	-	217.15	3.17	M+ACN+H	C8H17NO3	1.07	1.17	0.01
255	pos_628	Ile Ile	metab_5745	-	245.19	3.22	M+H, M+Na	C12H24N2O3	1.15	1.18	0.02

256	pos_656	Leu Leu	metab_6041	-	245.19	3.42	M+H, M+Na	C12H24N2O3	1.16	1.18	0.01
257	neg_5855	Isoleucyl-Isoleucine	metab_12800	-	243.17	3.68	M-H	C12H24N2O3	1.15	1.18	0.04
258	pos_102	(2S,3R)-2-Amino-3-[(2S)-2-amino-3-hydroxypropanoyl]oxybutanoic acid	metab_24	-	207.10	0.62	M+H, M+K, M+H-H2O	C7H14N2O5	1.05	1.18	0.04
259	pos_4851	Leu Phe Leu	metab_4172	-	392.25	5.10	M+H	C21H33N3O4	1.12	1.18	0.00
260	neg_2112	Allysine	metab_15407	1962-83-0;6665-12-9	349.16	2.69	2M+Hac-H	C6H11NO3	1.13	1.18	0.02
261	neg_1237	Xylobiose	metab_7921	6860-47-5	845.28	0.79	3M-H	C10H18O9	1.11	1.18	0.04
262	pos_5506	Glutathione	metab_5830	15718-51-1;70-18-8;27025-41-8	308.09	1.21	M+H	C10H17N3O6S	1.19	1.18	0.02
263	neg_7101	Gaboxadol	metab_14096	64603-91-4	185.06	1.96	M+FA-H	C6H8N2O2	1.08	1.18	0.03
264	pos_676	PGP(PGJ2/18:3(9Z,12Z,15Z))	metab_6251	-	903.44	3.55	M+H, M+Na	C44H72O15P2	1.20	1.18	0.01
265	pos_2251	(2S)-5-(Diaminomethylideneamino)-2-(hydroxyamino)pentanoic Acid	metab_1352	-	422.25	2.14	2M+ACN+H	C6H14N4O3	1.12	1.18	0.02
266	pos_6164	Val Ile	metab_5621	-	231.17	2.44	M+H	C11H22N2O3	1.16	1.18	0.01
267	pos_1537	Arg Ser	metab_591	-	262.15	0.58	M+H	C9H19N5O4	1.06	1.18	0.04
268	neg_6144	Perindopril	metab_13115	107133-36-8;82834-16-0	389.20	3.22	M+Na-2H	C19H32N2O5	1.27	1.18	0.01

269	pos_359	L-Dopa	metab_2822	59-92-7	198.08	1.60	M+H, M+NH4	C9H11NO4	1.10	1.18	0.00
270	pos_1319	Val Arg	metab_355	-	274.19	0.69	M+2H, M+H	C11H23N5O3	1.12	1.18	0.03
271	pos_6173	Hydroxyzine	metab_5631	68-88-2	375.19	2.43	M+H	C21H27ClN2O2	1.21	1.18	0.00
272	pos_5545	Estriol-16-Glucuronide	metab_4936	1852-50-2	429.19	3.40	M+H-2H2O	C24H32O9	1.09	1.18	0.01
273	neg_6183	Metipranolol	metab_13148	22664-55-7	330.17	3.18	M+Na-2H	C17H27NO4	1.26	1.18	0.02
274	pos_176	Asn Leu	metab_826	-	246.14	2.19	M+H, M+Na, M+H-H2O	C10H19N3O4	1.20	1.18	0.02
275	pos_1770	7-Epijasmonic acid	metab_838	-	233.11	1.00	M+Na	C12H18O3	1.10	1.18	0.02
276	pos_2517	Candoxatrilat	metab_1638	123898-42-0	422.21	2.67	M+Na	C20H33NO7	1.10	1.18	0.01
277	pos_7017	Met Arg	metab_6522	-	306.16	1.13	M+H	C11H23N5O3S	1.05	1.18	0.04
278	pos_3036	Phe Leu	metab_2210	-	279.17	3.90	M+H	C15H22N2O3	1.20	1.18	0.02
279	neg_5777	Pisumionoside	metab_12716	371113-06-3	449.20	3.80	M+FA-H	C19H32O9	1.16	1.18	0.01
280	pos_7471	Meclizine	metab_7005	569-65-3	432.22	0.59	M+ACN+H	C25H27ClN2	1.15	1.19	0.01
281	neg_2052	Valylmethionine	metab_8726	14486-09-0	247.11	2.58	M-H	C10H20N2O3S	1.18	1.19	0.03
282	neg_6072	Trandolaprilat	metab_13039	-	401.20	3.32	M-H	C22H30N2O5	1.28	1.19	0.01
283	pos_1893	3''-deamino-3''-oxonicotianamine	metab_968	-	319.14	1.30	M+NH4	C12H17N2O7-	1.17	1.19	0.01
284	pos_2294	(-)-Slaframine	metab_1396	20084-93-9	231.17	2.25	M+CH3OH+H	C10H18N2O2	1.18	1.19	0.01
285	neg_2980	Merodesmosine	metab_9705	17096-97-8	383.23	4.23	M-H2O-H	C18H34N4O6	1.23	1.19	0.01
286	pos_5906	Beta-Leucine	metab_5337	5699-54-7	326.21	2.81	2M+ACN+Na	C6H13NO2	1.17	1.19	0.00
287	neg_239	Gamma-Glutamylvaline	metab_9075	2746-34-1	245.11	1.23	M-H2O-H, M-H	C10H18N2O5	1.27	1.19	0.03
288	pos_6738	Tazolol	metab_6228	-	474.22	1.79	2M+ACN+H	C9H16N2O2S	1.18	1.19	0.00

289	neg_2365	Ramiprilat	metab_9050	87269-97-4	387.19	3.10	M-H	C21H28N2O5	1.31	1.19	0.01
290	pos_5019	Asp Phe Leu	metab_4359	-	394.20	4.54	M+H	C19H27N3O6	1.02	1.19	0.01
291	neg_2428	Cinncassiol D2 glucoside	metab_9115	-	529.26	3.18	M-H	C26H42O11	1.27	1.19	0.01
292	neg_2174	Monopropionylcadaverine	metab_8847	-	157.13	2.80	M-H	C8H18N2O	1.11	1.19	0.02
293	neg_1527	Histidinyl-Leucine	metab_8201	7763-65-7	267.15	1.48	M-H	C12H20N4O3	1.27	1.19	0.03
294	pos_5261	Leu Trp	metab_4624	-	318.18	3.99	M+H	C17H23N3O3	1.10	1.19	0.02
295	neg_2242	Ribalinium	metab_8921	6883-22-3	335.14	2.90	M+FA-H	C16H20NO4+	1.28	1.19	0.01
296	pos_2056	3-Hydroxy-L-proline	metab_1142	876067.00	304.15	1.82	2M+ACN+H	C5H9NO3	1.12	1.19	0.01
297	neg_2339	Delta-Hydroxylysylnorleucine	metab_9023	-	272.16	3.06	M-H2O-H	C12H25N3O5	1.31	1.19	0.01
298	pos_6041	Dactimicin	metab_5487	73196-97-1	496.29	2.60	M+ACN+Na	C18H36N6O6	1.16	1.19	0.00
299	neg_7930	Metanephrine	metab_14892	5001-33-2	218.08	0.58	M+Na-2H	C10H15NO3	1.18	1.19	0.01
300	pos_6788	Methionyl-Serine	metab_6278	-	237.09	1.64	M+H	C8H16N2O4S	1.15	1.19	0.01
301	pos_5300	Val Met Ile	metab_4668	-	362.21	3.90	M+H	C16H31N3O4S	1.14	1.19	0.00
302	pos_1257	Asn Thr	metab_286	-	234.11	0.62	M+H, M+K	C8H15N3O5	1.17	1.19	0.01
303	pos_7415	Val His	metab_6944	-	255.14	0.63	M+H	C11H18N4O3	1.18	1.19	0.01
304	pos_5266	Kainic acid	metab_4629	487-79-6	277.12	3.98	M+ACN+Na	C10H15NO4	1.10	1.19	0.01
305	pos_2828	N-Acetylserotonin	metab_1980	1210-83-9	251.14	3.35	M+CH3OH+H	C12H14N2O2	1.04	1.19	0.01
306	neg_8120	Histidinyl-Lysine	metab_15098	37700-85-9	282.16	0.50	M-H	C12H21N5O3	1.19	1.19	0.02
307	neg_5154	Glu-val-phe	metab_12039	-	392.18	4.98	M-H	C19H27N3O6	1.11	1.19	0.04
308	pos_5794	N(2)-phenylacetyl-L-glutaminate	metab_5212	-	306.14	3.00	M+ACN+H	C13H16N2O4	1.09	1.19	0.02
309	neg_1835	Edivoxetine	metab_8499	-	374.16	2.10	M+Cl	C18H26FNO4	1.35	1.19	0.00

310	neg_1022	Lysylarginine	metab_7702	29586-66-1	301.20	0.49	M-H	C12H26N6O3	1.24	1.19	0.01
311	neg_6440	Leucyl-Valine	metab_13414	-	229.16	2.87	M-H	C11H22N2O3	1.26	1.19	0.02
312	neg_2782	N-[(3a,5b,7a)-3-hydroxy-24-oxo-7-(sulfooxy)cholan-24-yl]-Glycine	metab_9491	67030-55-1	574.27	3.84	M+FA-H	C26H43NO8S	1.19	1.20	0.02
313	neg_7048	Valylcysteine	metab_14038	160070-15-5	219.08	2.04	M-H	C8H16N2O3S	1.20	1.20	0.03
314	pos_1934	2-Keto-6-acetamidocaproate	metab_1013	-	205.12	1.50	M+NH4	C8H13NO4	1.21	1.20	0.02
315	pos_420	Glutaminyltyrosine	metab_3471	457662-02-1	342.17	1.94	M+NH4, M+CH3OH+H	C14H19N3O5	1.13	1.20	0.02
316	pos_2391	Asn Phe	metab_1500	-	280.13	2.44	M+H	C13H17N3O4	1.10	1.20	0.03
317	pos_6571	Herculin	metab_6054	-	274.21	1.95	M+Na	C16H29NO	1.21	1.20	0.00
318	pos_4666	Zolmitriptan	metab_3970	139264-17-8	616.36	5.62	2M+ACN+H	C16H21N3O2	1.00	1.20	0.03
319	pos_2033	Glycyl-Methionine	metab_1121	-	207.08	1.72	M+H	C7H14N2O3S	1.11	1.20	0.03
320	pos_167	Ala Pro Val	metab_729	-	318.20	2.03	M+H, 2M+K, M+CH3OH+H	C13H23N3O4	1.22	1.20	0.00
321	pos_1849	Ile Arg	metab_920	-	288.20	1.16	M+H	C12H25N5O3	1.29	1.20	0.00
322	neg_6652	Ala-Leu	metab_13635	3303-34-2	201.12	2.58	M-H	C9H18N2O3	1.29	1.20	0.02
323	neg_3269	Cholylphenylalanine	metab_10020	-	592.30	4.91	M+K-2H	C33H49NO6	1.34	1.20	0.00
324	neg_5352	LysoPE(0:0/20:1(11Z))	metab_12255	-	528.30	4.51	M+Na-2H	C25H50NO7P	1.29	1.20	0.01

325	pos_5415	Ile Phe	metab_4794	-	279.17	3.66	M+H	C15H22N2O3	1.13	1.20	0.03
326	pos_528	1-Octanesulfonic acid	metab_4644	-	389.20	2.49	M+ACN+Na, 2M+H	C8H18O3S	1.24	1.20	0.00
327	pos_583	Ile Leu	metab_5252	-	245.19	2.91	M+H, M+Na	C12H24N2O3	1.19	1.20	0.02
328	pos_5601	Ceanothine E	metab_4999	23926-98-9	632.32	3.31	M+ACN+Na	C34H40N4O4	1.10	1.20	0.01
329	pos_7424	Dihydrothymine	metab_6954	19140-80-8;696-04-8	257.12	0.62	2M+H	C5H8N2O2	1.14	1.20	0.03
330	neg_1847	L-Leucyl-L-Alanine	metab_8512	7298-84-2	201.12	2.11	M-H	C9H18N2O3	1.30	1.20	0.01
331	neg_7798	Hydroxyprolyl-Gamma-glutamate	metab_14764	-	304.12	0.69	M+FA-H	C10H17N3O5	1.17	1.20	0.04
332	pos_2017	Tyrosyl-Gamma-glutamate	metab_1104	-	310.14	1.68	M+H	C14H19N3O5	1.11	1.20	0.03
333	pos_5174	Golotimod	metab_4529	-	316.13	4.18	M+H-H2O	C16H19N3O5	1.09	1.20	0.00
334	pos_204	Lys His	metab_1125	-	284.17	0.48	M+2H, M+H	C12H21N5O3	1.16	1.20	0.01
335	pos_5774	Deferoxamine	metab_5190	70-51-9	543.35	3.03	M+H-H2O	C25H48N6O8	1.16	1.20	0.01
336	neg_953	Abeado	metab_15353	-	370.16	4.21	M+Na-2H, 2M+FA-H	C15H23N7O3	1.15	1.20	0.03
337	neg_6330	Pexacerfont	metab_13296	-	361.17	3.01	M+Na-2H	C18H24N6O	1.28	1.20	0.00
338	neg_2710	Sulfinalol	metab_9414	-	376.16	3.71	M-H	C20H27NO4S	1.16	1.20	0.01
339	neg_3476	Goshonoside F4	metab_10246	90851-27-7	629.35	5.55	M-H	C32H54O12	1.21	1.20	0.03
340	neg_1905	Valylvaline	metab_8569	3918-94-3	215.14	2.29	M-H	C10H20N2O3	1.28	1.20	0.03
341	pos_2795	N1-trans-Feruloylagmatine	metab_1943	-	651.30	3.28	2M+K	C15H22N4O3	1.21	1.20	0.00
342	pos_3175	N-(8-Amino-1-carboxyoctyl)-alanyl-proline	metab_2363	-	399.26	4.34	M+ACN+H	C17H31N3O5	1.21	1.20	0.00
343	pos_2102	Thr Met	metab_1193	-	251.11	1.89	M+H	C9H18N2O4S	1.17	1.20	0.01

344	neg_648	LysoPA(i-13:0/0:0)	metab_13452	-	389.17	2.41	M+Na-2H, M+K-2H	C16H33O7P	1.33	1.20	0.01
345	pos_2446	2,6-Diamino-5-hydroxyhexanoic acid	metab_1561	1190-94-9	347.19	2.53	2M+Na	C6H14N2O3	1.23	1.20	0.00
346	pos_7485	Arg Gln	metab_7020	-	303.18	0.58	M+H	C11H22N6O4	1.11	1.20	0.04
347	neg_5989	Val-pro-pro	metab_12946	-	356.18	3.47	M+FA-H	C15H25N3O4	1.17	1.20	0.01
348	pos_1894	Ile Asp Lys	metab_969	-	375.22	1.30	M+H	C16H30N4O6	1.20	1.20	0.01
349	pos_537	Thr Glu Leu	metab_4744	-	362.19	2.59	M+H, 2M+Na	C15H27N3O7	1.32	1.20	0.00
350	neg_7310	Leucyl-Serine	metab_14290	-	217.12	1.52	M-H	C9H18N2O4	1.36	1.21	0.01
351	neg_2263	Erythrohydrobupropion	metab_8944	-	541.26	2.93	2M+Hac-H	C13H20ClNO	1.28	1.21	0.00
352	pos_3189	Madecassoside	metab_2378	34540-22-2	499.25	4.38	M+H+Na	C48H78O20	1.14	1.21	0.02
353	pos_7664	Arg Lys	metab_7217	-	303.21	0.51	M+H	C12H26N6O3	1.22	1.21	0.01
354	pos_2869	Glutamylleucine	metab_2024	5969-52-8	243.13	3.44	M+H-H2O	C11H20N2O5	1.19	1.21	0.01
355	neg_5578	Lefamulin	metab_12500	-	506.30	4.13	M-H	C28H45NO5S	1.38	1.21	0.00
356	pos_1185	Val Pro Asp	metab_206	-	330.17	2.02	M+H, M+ACN+H	C14H23N3O6	1.15	1.21	0.01
357	neg_1460	(4S,6R)-p-Mentha-1,8-diene-6,7-diol 7-glucoside	metab_8134	402593-54-8	351.14	1.24	M+Na-2H	C16H26O7	1.20	1.21	0.02
358	pos_1538	Asn Arg	metab_592	-	289.16	0.58	M+H	C10H20N6O4	1.20	1.21	0.00
359	neg_7325	Leucyl-Lysine	metab_14305	-	258.18	1.48	M-H	C12H25N3O3	1.34	1.21	0.02
360	pos_2009	L-Lysine	metab_1095	10098-89-2;56-87-1	129.10	1.65	M+H-H2O	C6H14N2O2	1.11	1.21	0.02
361	pos_6929	His Ala Val	metab_6431	-	326.18	1.33	M+H	C14H23N5O4	1.26	1.21	0.00

362	pos_1635	Lys Ser	metab_692	-	234.14	0.76	M+H	C9H19N3O4	1.09	1.21	0.05
363	neg_2043	Asparaginyl-Leucine	metab_8716	-	244.13	2.57	M-H	C10H19N3O4	1.34	1.21	0.01
364	neg_6071	Xanthine amine congener	metab_13038	-	463.19	3.32	M+Cl	C21H28N6O4	1.30	1.21	0.00
365	pos_6515	Indicine-N-oxide	metab_5993	-	329.18	2.02	2M+3H2O+2H	C15H25NO6	1.13	1.21	0.02
366	pos_2713	Leu Ile	metab_1853	-	245.19	3.11	M+H	C12H24N2O3	1.20	1.21	0.01
367	neg_5624	Protoveratrine	metab_12550	-	814.40	4.05	M+Na-2H	C41H63NO14	1.25	1.21	0.03
368	pos_6129	Isoleucylhydroxyproline	metab_5582	90965-80-3	286.18	2.49	M+ACN+H	C11H20N2O4	1.23	1.21	0.00
369	pos_5565	Milbemycin beta3	metab_4958	-	517.30	3.37	M+Na	C31H42O5	1.23	1.21	0.00
370	neg_3443	Pravastatin	metab_10211	81093-37-0	405.22	5.49	M-H2O-H	C23H36O7	1.26	1.21	0.04
371	pos_6979	2-Hydroxyphenethylamine	metab_6484	7568-93-6	338.18	1.18	2M+ACN+Na	C8H11NO	1.14	1.21	0.04
372	neg_824	Ile Ser Val Asp	metab_15211	-	431.22	3.36	M-H2O-H, M-H	C18H32N4O8	1.38	1.21	0.02
373	pos_6351	Xestoaminol C	metab_5824	-	274.21	2.18	M+2Na-H	C14H31NO	1.16	1.22	0.02
374	neg_6634	Glutaminylisoleucine	metab_13616	86416-45-7	258.15	2.60	M-H	C11H21N3O4	1.37	1.22	0.01
375	pos_7188	Ser Gln	metab_6701	-	234.11	0.86	M+H	C8H15N3O5	1.16	1.22	0.01
376	neg_341	Val Ala Ser Asp	metab_10175	-	389.17	2.08	M-H2O-H, M-H	C15H26N4O8	1.32	1.22	0.01
377	neg_238	Ethyl 2-hydroxy-3-(3-indolyl)propanoate glucoside	metab_9066	143884-93-9	394.15	1.16	M-H2O-H, M-H	C19H25NO8	1.32	1.22	0.01
378	neg_334	Histamine	metab_10099	51-45-6	110.07	0.56	M-H, M+FA-H	C5H9N3	1.35	1.22	0.01

379	pos_2062	Leu Lys Glu	metab_1149	-	389.24	1.83	M+H	C17H32N4O6	1.16	1.22	0.02
380	neg_5653	LysoPA(16:0/0:0)	metab_12582	-	431.22	4.00	M+Na-2H	C19H39O7P	1.29	1.22	0.01
381	neg_293	Hydroxyprolyl-Lysine	metab_9652	-	258.15	1.82	M-H2O-H, M-H	C11H21N3O4	1.44	1.22	0.01
382	pos_6876	Oxdralazine	metab_6372	-	444.28	1.52	2M+NH4	C8H15N5O2	1.32	1.22	0.00
383	neg_7876	Alanyl-Gamma-glutamate	metab_14839	-	216.10	0.62	M-H	C8H15N3O4	1.28	1.22	0.01
384	neg_3138	24-Acetyl-25-cinnamoylvulgaroside	metab_9877	172616-89-6	589.32	4.55	M-H2O-H	C36H48O8	1.23	1.22	0.03
385	neg_6112	Benzamide, 3-(3-((2-(3,4-dihydroxyphenyl)ethyl)amino)butyl)-	metab_13082	-	349.15	3.25	M+Na-2H	C19H24N2O3	1.19	1.22	0.03
386	neg_4874	3-(3-Indolyl)-2-oxopropanoic acid	metab_11729	392-12-1	202.05	5.64	M-H	C11H9NO3	1.13	1.22	0.02
387	neg_1994	Serylleucine	metab_8663	6665-16-3	217.12	2.50	M-H	C9H18N2O4	1.35	1.22	0.03
388	neg_5968	Dual-release hydrocortisone	metab_12923	-	471.26	3.50	M+FA-H	C22H38N2O4S	1.32	1.22	0.01
389	neg_2592	P-Octopamine	metab_9287	104-14-3	458.23	3.48	3M-H	C8H11NO2	1.32	1.22	0.01
390	neg_6260	Metoprolol	metab_13220	37350-58-6;51384-51-1	288.16	3.11	M+Na-2H	C15H25NO3	1.42	1.22	0.01
391	pos_1665	Ser Arg	metab_724	-	262.15	0.81	M+H	C9H19N5O4	1.16	1.22	0.02
392	neg_5244	Corticosterone	metab_12137	50-22-6	391.21	4.77	M+FA-H	C21H30O4	1.25	1.22	0.03
393	neg_5037	PC(18:1(12Z)-O(9S,10R)/2:0)	metab_11909	-	558.31	5.34	M-H2O-H	C28H52NO9P	1.42	1.22	0.00
394	pos_5369	NH-DVal(NMe)-Val-OMe	metab_4743	-	245.19	3.75	M+H	C12H24N2O3	1.27	1.22	0.01

395	pos_2270	Leu Val Thr	metab_1372	-	332.22	2.21	M+H	C15H29N3O5	1.25	1.22	0.00
396	neg_5902	Voacamine	metab_12852	3371-85-5	739.36	3.59	M+Cl	C43H52N4O5	1.34	1.22	0.00
397	neg_6572	Ser Thr Thr Ala Val	metab_13551	-	476.24	2.69	M-H	C19H35N5O9	1.42	1.22	0.00
398	pos_2594	Thyrotropin-releasing factor	metab_1721	-	395.20	2.83	M+CH3OH+H	C16H22N6O4	1.17	1.22	0.01
399	neg_2273	2',3'-Dideoxyuridine	metab_8955	-	423.15	2.95	2M-H	C9H12N2O4	1.35	1.22	0.00
400	neg_3534	LysoPG(16:0/0:0)	metab_10310	116870-26-9	519.25	5.63	M+Cl	C22H45O9P	1.25	1.23	0.01
401	pos_1610	Leu Arg	metab_668	-	288.20	0.69	M+H	C12H25N5O3	1.28	1.23	0.00
402	neg_433	N-[(2Z)-2-Methoxyiminopropyl]-7H-purin-6-amine	metab_11155	-	439.21	5.63	M-H, 2M-H	C9H12N6O	1.30	1.23	0.03
403	pos_6403	Leucyl-Glutamine	metab_5878	-	242.15	2.11	M+H-H2O	C11H21N3O4	1.16	1.23	0.01
404	pos_6668	Val Glu Glu	metab_6159	-	376.17	1.87	M+H	C15H25N3O8	1.25	1.23	0.00
405	pos_5745	PS(18:1(11Z)/18:2(9Z,12Z))	metab_5158	-	415.75	3.08	M+2Na	C42H76NO10P	1.21	1.23	0.02
406	pos_5083	Leu Pro Phe	metab_4428	-	376.22	4.39	M+H	C20H29N3O4	1.25	1.23	0.00
407	pos_7079	Val Gln	metab_6587	-	246.14	1.02	M+H	C10H19N3O4	1.26	1.23	0.01
408	neg_6437	Gln Asp Leu Glu	metab_13411	-	502.22	2.87	M-H	C20H33N5O10	1.34	1.23	0.00
409	pos_6182	Val Gly Val	metab_5641	-	274.18	2.42	M+H	C12H23N3O4	1.28	1.23	0.01
410	pos_6370	Thr Leu	metab_5843	-	233.15	2.16	M+H	C10H20N2O4	1.21	1.23	0.03
411	neg_5831	Cis-and trans-Ethyl 2,4-dimethyl-1,3-dioxolane-2-acetate	metab_12774	-	421.21	3.72	2M+FA-H	C9H16O4	1.24	1.23	0.02

412	neg_6146	Etodolac	metab_13117	41340-25-4	308.13	3.22	M+Na-2H	C17H21NO3	1.27	1.23	0.03
413	neg_2832	Moxifloxacin	metab_9547	151096-09-2;354812-41-2	847.35	3.93	2M+FA-H	C21H24FN3O4	1.25	1.23	0.03
414	pos_5961	Dihydrodigoxin	metab_5398	1241013.00	403.22	2.72	M+H+Na	C41H66O14	1.25	1.23	0.01
415	pos_2835	Herbimycin a	metab_1988	70563-58-5	638.30	3.36	M+ACN+Na	C30H42N2O9	1.31	1.23	0.00
416	neg_2663	Levetiracetam	metab_9363	102767-28-2	399.23	3.60	2M+Hac-H	C8H14N2O2	1.25	1.23	0.05
417	neg_7270	D[-Arg-2]KYOTORPHAN	metab_14254	-	336.17	1.63	M-H	C15H23N5O4	1.28	1.23	0.01
418	neg_5574	Carbuterol	metab_12496	-	312.16	4.14	M+FA-H	C13H21N3O3	1.15	1.23	0.04
419	pos_5144	Verdamicin	metab_4496	-	444.28	4.26	M+H-H2O	C20H39N5O7	1.24	1.23	0.00
420	pos_2448	Flibanserin	metab_1563	-	408.20	2.53	M+NH4	C20H21F3N4O	1.22	1.23	0.01
421	neg_7343	Lysyltyrosine	metab_14324	35978-98-4	308.16	1.42	M-H	C15H23N3O4	1.28	1.23	0.03
422	neg_3170	21-Deoxycortisol	metab_9912	641-77-0	391.21	4.63	M+FA-H	C21H30O4	1.21	1.23	0.02
423	neg_6036	Dihydroneopine	metab_12999	-	322.14	3.39	M+Na-2H	C18H23NO3	1.26	1.23	0.04
424	neg_1443	Leucyl-Arginine	metab_8117	-	286.19	1.17	M-H	C12H25N5O3	1.37	1.23	0.00
425	pos_6332	Pro Gln Val	metab_5803	-	343.20	2.21	M+H	C15H26N4O5	1.24	1.23	0.01
426	pos_4849	Ile Ile Ile	metab_4169	-	358.27	5.11	M+H	C18H35N3O4	1.25	1.23	0.00
427	pos_6787	Asp Ile Lys	metab_6277	-	375.22	1.64	M+H	C16H30N4O6	1.30	1.23	0.01
428	neg_2685	Phenylalanylmethionine	metab_9386	15080-84-9	295.11	3.66	M-H	C14H20N2O3S	1.25	1.23	0.02
429	pos_6483	2-Deoxystreptamine	metab_5957	2037-48-1	347.19	2.03	2M+Na	C6H14N2O3	1.28	1.23	0.00
430	neg_3121	Glycylphenylalanylleucylglycine	metab_9859	-	391.20	4.50	M-H	C19H28N4O5	1.24	1.23	0.01

431	pos_1140	Ala Asp Leu	metab_157	-	318.17	2.49	M+H-H2O, M+H	C13H23N3O6	1.32	1.24	0.01
432	neg_1892	Beta-Funaltrexamine	metab_8556	72782-05-9	489.18	2.24	M+Cl	C25H30N2O6	1.45	1.24	0.00
433	neg_720	Gly-pro-arg-pro	metab_14194	-	460.20	2.83	M-H2O-H, M+Cl	C18H31N7O5	1.33	1.24	0.03
434	neg_1560	Asparaginyl-Valine	metab_8233	-	230.11	1.60	M-H	C9H17N3O4	1.43	1.24	0.01
435	neg_5301	Subaphylline	metab_12199	501-13-3	573.29	4.63	2M+FA-H	C14H20N2O3	1.38	1.24	0.02
436	pos_2660	Dehydroepiandrosterone sulfate	metab_1795	651-48-9	401.20	2.99	M+CH3OH+H	C19H28O5S	1.27	1.24	0.01
437	neg_7322	Serylvaline	metab_14302	51782-06-0	203.10	1.49	M-H	C8H16N2O4	1.40	1.24	0.03
438	neg_3221	Tryptophyl-Isoleucine	metab_9968	-	316.17	4.78	M-H	C17H23N3O3	1.33	1.24	0.02
439	neg_3009	Triterpenoid	metab_9738	-	573.29	4.28	M+Na-2H	C30H48O7S	1.35	1.24	0.02
440	neg_5581	Cabastine	metab_12504	-	885.43	4.12	2M+FA-H	C26H29FN2O2	1.31	1.24	0.03
441	pos_5501	Vigabatrin	metab_4888	60643-86-9	300.19	3.49	2M+ACN+H	C6H11NO2	1.25	1.24	0.00
442	pos_6648	Asp Lys Leu	metab_6137	-	375.22	1.90	M+H	C16H30N4O6	1.32	1.24	0.01
443	pos_6215	L-Norleucine	metab_5678	327-57-1	173.13	2.37	M+ACN+H	C6H13NO2	1.24	1.24	0.00
444	neg_5229	(p-SCN-Bn)-dota	metab_12121	-	596.20	4.81	M+FA-H	C24H33N5O8S	1.35	1.24	0.00
445	pos_3174	Crotetamide	metab_2362	-	259.20	4.34	M+CH3OH+H	C12H22N2O2	1.26	1.24	0.00
446	neg_2504	S-Farnesyl cysteine	metab_9192	68000-92-0	360.18	3.29	M+Cl	C18H31NO2S	1.44	1.24	0.01
447	neg_787	1-(3-Methyl-2-butenoyl)-6-apiosylglucose	metab_14835	467242-32-6	393.14	3.16	M-H2O-H, M-H	C16H26O11	1.34	1.24	0.01

448	neg_5331	Rivenprost	metab_12232	-	431.19	4.57	M-H2O-H	C24H34O6S	1.21	1.24	0.04
449	pos_5973	Epothilone A	metab_5411	152044-53-6	511.29	2.69	M+NH4	C26H39NO6S	1.27	1.25	0.01
450	neg_5721	Neopine	metab_12656	467-14-1	657.32	3.88	2M+Hac-H	C18H21NO3	1.35	1.25	0.03
451	pos_2502	Ser Gly Phe	metab_1622	-	310.14	2.63	M+H	C14H19N3O5	1.27	1.25	0.00
452	neg_5936	Ketotrilostane	metab_12889	-	348.16	3.55	M+Na-2H	C20H25NO3	1.37	1.25	0.00
453	pos_2551	Ultram	metab_1675	27203-92-5	286.18	2.75	M+Na	C16H25NO2	1.34	1.25	0.01
454	pos_5661	1-Octen-3-yl primeveroside	metab_5065	209863-01-4	423.22	3.21	M+H	C19H34O10	1.17	1.25	0.03
455	neg_4888	O-Desmethylverapamil (D-702)	metab_11744	-	485.26	5.63	M+FA-H	C26H36N2O4	1.32	1.25	0.02
456	pos_3634	GPEtn(16:1/22:5)	metab_2869	-	764.52	6.62	M+H	C43H74NO8P	1.75	1.25	0.01
457	neg_2901	Penitrem E	metab_9622	78213-66-8	598.32	4.07	M-H	C37H45NO6	1.39	1.25	0.02
458	neg_5698	Moroxydine	metab_12629	-	387.23	3.93	2M+FA-H	C6H13N5O	1.41	1.25	0.01
459	neg_5556	3-(Benzylamino)-3-methylbutanoic Acid	metab_12476	-	413.24	4.16	2M-H	C12H17NO2	1.37	1.25	0.04
460	neg_5682	PC(20:5(7Z,9Z,11E,13E,17Z)-3OH(5,6,15)/2:0)	metab_12612	-	630.31	3.95	M-H	C30H50NO11P	1.33	1.25	0.02
461	neg_5548	CPA(18:0/0:0)	metab_12467	-	441.24	4.18	M+Na-2H	C21H41O6P	1.32	1.25	0.01
462	pos_2075	Alanylmethionine	metab_1162	14486-05-6	221.10	1.86	M+H	C8H16N2O3S	1.25	1.25	0.02
463	pos_6330	MG(5-iso PGF2VI/0:0/0:0)	metab_5801	-	442.28	2.21	M+ACN+H	C21H36O7	1.29	1.25	0.01
464	neg_2456	Leukotriene-D4	metab_9142	-	516.23	3.21	M+Na-2H	C25H39N2O6S-	1.39	1.25	0.02
465	neg_7037	Glutaminylmethionine	metab_14027	114659-59-5	276.10	2.06	M-H	C10H19N3O4S	1.27	1.25	0.02

466	neg_915	N-alpha-Acetyl-L-citrulline	metab_15311	33965-42-3	433.21	3.96	2M-H, 2M+Hac-H	C8H15N3O4	1.34	1.25	0.01
467	pos_5426	L-Hypoglycin A	metab_4806	156-56-9	300.19	3.63	2M+NH4	C7H11NO2	1.25	1.25	0.00
468	pos_1993	1-(3-Pyridinyl)-1,4-butanediol	metab_1078	76014-83-0	398.20	1.62	2M+ACN+Na	C9H13NO2	1.31	1.25	0.00
469	neg_5820	H-D-Tyr-val-gly-OH	metab_12762	-	318.15	3.74	M-H2O-H	C16H23N3O5	1.32	1.25	0.02
470	neg_5106	Janthitrem E	metab_11986	90986-50-8	584.34	5.12	M-H2O-H	C37H49NO6	1.46	1.25	0.01
471	pos_6352	Aminocaproic acid	metab_5825	60-32-2	173.13	2.18	M+ACN+H	C6H13NO2	1.18	1.25	0.02
472	pos_104	Glutaminylserine	metab_46	5875-40-1	275.13	0.62	M+H-H2O, M+ACN+H, M+H-2H2O	C8H15N3O5	1.30	1.25	0.00
473	neg_5976	3-Hydroxy-beta-ionol 3-[glucosyl-(1->6)-glucoside]	metab_12932	137606-56-5	555.24	3.48	M+Na-2H	C25H42O12	1.44	1.25	0.00
474	pos_6600	Aspartyl-Lysine	metab_6086	5891-51-0	303.17	1.94	M+ACN+H	C10H19N3O5	1.25	1.25	0.01
475	neg_5100	Silux	metab_11980	-	547.21	5.14	M+Cl	C29H36O8	1.35	1.25	0.02
476	neg_2797	Leukotriene E3	metab_9507	79494-05-6	486.26	3.87	M+FA-H	C23H39NO5S	1.41	1.25	0.01
477	pos_1145	Ac-Pro-Gly-Pro-OH	metab_162	-	344.18	2.18	M+CH3OH+H, 2M+H	C14H21N3O5	1.26	1.25	0.02
478	neg_2779	Perindoprilat	metab_9487	-	375.17	3.84	M+Cl	C17H28N2O5	1.38	1.25	0.01
479	pos_5944	Asp Ala Phe	metab_5379	-	352.15	2.75	M+H	C16H21N3O6	1.23	1.25	0.01
480	neg_5426	Pangamic acid	metab_12336	11006-56-7	473.23	4.38	M+K-2H	C20H40N2O8	1.32	1.25	0.01
481	pos_2895	Cyclo(L-Phe-L-Pro)	metab_2053	3705-26-8	277.15	3.51	M+CH3OH+H	C14H16N2O2	1.24	1.25	0.00

482	pos_5007	Leucyl-Glutamate	metab_4346	-	243.13	4.59	M+H-H2O	C11H20N2O5	1.17	1.25	0.01
483	neg_6590	Threonylisoleucine	metab_13569	129050-49-3	231.13	2.68	M-H	C10H20N2O4	1.39	1.25	0.03
484	neg_2831	PC(2:0/PGJ2)	metab_9546	-	636.29	3.93	M+Na-2H	C30H50NO10P	1.42	1.26	0.02
485	neg_5761	Beta-Zearalenol	metab_15409	71030-11-0;36455-72-8	699.33	3.82	2M+Hac-H	C18H24O5	1.39	1.26	0.01
486	pos_2947	Mesobilirubinogen	metab_2111	14684-37-8	593.33	3.65	M+H	C33H44N4O6	1.31	1.26	0.00
487	neg_6688	Bursin	metab_13668	-	360.18	2.52	M+Na-2H	C14H25N7O3	1.37	1.26	0.02
488	pos_5242	(1S,2S,4R)-p-Menth-8-ene-1,2,10-triol 2-glucoside	metab_4603	378254-11-6	381.21	4.03	M+CH3OH+H	C16H28O8	1.24	1.26	0.00
489	pos_3167	Forskolin	metab_7659	66575-29-9;3413-15-8	393.22	4.32	M+H-H2O	C22H34O7	1.26	1.26	0.01
490	neg_6203	Imidaprilat	metab_13168	89371-44-8	376.15	3.16	M-H	C18H23N3O6	1.39	1.26	0.00
491	pos_2879	Phe Ile	metab_2035	-	279.17	3.48	M+H	C15H22N2O3	1.21	1.26	0.04
492	pos_6384	Cortolone-3-glucuronide	metab_5856	56162-46-0	584.30	2.13	M+ACN+H	C27H42O11	1.36	1.26	0.00
493	pos_6016	Asp Glu Leu	metab_5459	-	376.17	2.63	M+H	C15H25N3O8	1.22	1.26	0.01
494	neg_7856	Threonylglutamine	metab_14823	96337-79-0	246.11	0.64	M-H	C9H17N3O5	1.35	1.26	0.02
495	neg_2757	Fumonisin B2	metab_9464	116355-84-1	686.37	3.79	M-H2O-H	C34H59NO14	1.43	1.26	0.02
496	pos_3127	Leu Asp Ile	metab_2311	-	360.21	4.20	M+H	C16H29N3O6	1.31	1.26	0.01
497	neg_2796	Velagliflozin	metab_9506	-	376.15	3.87	M-H2O-H	C23H25NO5	1.29	1.26	0.02
498	neg_2362	Toosendanin	metab_9047	-	619.24	3.10	M+FA-H	C30H38O11	1.45	1.26	0.01
499	pos_5590	N-Methoxysuccinyl-Ala-Ala-Pro-Val	metab_4986	-	534.26	3.32	M+ACN+Na	C21H34N4O8	1.35	1.26	0.00
500	pos_3155	Pimaricin	metab_2342	7681-93-8	666.31	4.28	M+H	C33H47NO13	1.25	1.26	0.01

501	neg_5130	N-Myristoyl Tryptophan	metab_12013	-	413.28	5.04	M-H	C25H38N2O3	1.46	1.26	0.01
502	pos_3047	Leu Pro Ile	metab_2222	-	342.24	3.93	M+H	C17H31N3O4	1.26	1.26	0.01
503	neg_5448	O-Phenolsulfonic acid	metab_12357	-	172.99	4.34	M-H	C6H6O4S	1.44	1.26	0.00
504	pos_2987	Ile Trp	metab_2154	-	318.18	3.77	M+H	C17H23N3O3	1.22	1.26	0.03
505	neg_3033	Cyclofoxy	metab_9761	-	390.17	4.32	M+FA-H	C20H24FNO3	1.32	1.26	0.01
506	neg_7252	Valylglutamine	metab_14234	42854-54-6	244.13	1.68	M-H	C10H19N3O4	1.45	1.26	0.02
507	pos_556	1,2,10-Trihydroxydihydro-trans-linalyl oxide 7-O-beta-D-glucopyranoside	metab_4952	219814-37-6	415.22	2.72	M+H-H2O, M+CH3OH+H	C16H30O10	1.28	1.27	0.03
508	pos_3088	CDP-DG(i-12:0/i-24:0)	metab_2267	-	516.78	4.07	M+H+Na	C48H89N3O15P2	1.32	1.27	0.01
509	pos_2360	Ser Ile Met	metab_1467	-	350.17	2.38	M+H	C14H27N3O5S	1.34	1.27	0.00
510	neg_5503	LysoPE(16:1(9Z)/0:0)	metab_12418	-	472.24	4.25	M+Na-2H	C21H42NO7P	1.35	1.27	0.03
511	neg_5596	PA(PGF2alpha/10:0)	metab_12519	-	699.32	4.10	M+K-2H	C33H59O11P	1.47	1.27	0.00
512	neg_1651	Ritodrine	metab_8314	26652-09-5	308.13	1.84	M+Na-2H	C17H21NO3	1.40	1.27	0.02
513	pos_6712	Histidinal	metab_6203	23784-15-8	342.17	1.83	2M+ACN+Na	C6H9N3O	1.25	1.27	0.01
514	neg_5246	Acetophenazine	metab_12139	2751-68-0	392.18	4.77	M-H2O-H	C23H29N3O2S	1.23	1.27	0.04
515	neg_7387	Terazosin	metab_14366	63590-64-7	408.16	1.28	M+Na-2H	C19H25N5O4	1.34	1.27	0.01
516	neg_5619	3-Methoxytyramine	metab_12545	554-52-9	500.27	4.06	3M-H	C9H13NO2	1.43	1.27	0.01

517	pos_6843	Asn Leu Lys	metab_6336	-	374.24	1.60	M+H	C16H31N5O5	1.33	1.27	0.00
518	neg_5853	LysoPE(18:2(9Z,12Z)/0:0)	metab_12798	85046-18-0	498.26	3.68	M+Na-2H	C23H44NO7P	1.40	1.27	0.01
519	neg_5290	Methylprednisolone hemisuccinate	metab_12186	-	455.20	4.66	M-H2O-H	C26H34O8	1.35	1.27	0.03
520	pos_4924	Pumiliotoxin A	metab_4253	67054-00-6	330.24	4.85	M+Na	C19H33NO2	1.28	1.27	0.00
521	pos_3129	Asebotoxin	metab_2313	-	457.22	4.20	M+2Na-H	C22H36O7	1.32	1.27	0.00
522	pos_2369	Phe Leu Arg	metab_1476	-	435.27	2.40	M+H	C21H34N6O4	1.36	1.27	0.00
523	neg_1637	Arginylisoleucine	metab_8300	62632-70-6	286.19	1.81	M-H	C12H25N5O3	1.52	1.27	0.01
524	neg_3546	Chenodeoxycholylmethionine	metab_10323	-	568.34	5.64	M+FA-H	C29H49NO5S	1.44	1.27	0.00
525	neg_906	Kukoamine C	metab_15301	-	529.30	3.88	M-H2O-H, M-H	C28H42N4O6	1.39	1.27	0.01
526	neg_1620	Xenognosin A	metab_8286	76907-79-4	277.09	1.74	M+Na-2H	C16H16O3	1.40	1.27	0.04
527	neg_6110	Vanillylamine	metab_13080	1196-92-5	458.23	3.25	3M-H	C8H11NO2	1.50	1.27	0.00
528	neg_3319	1-Nonadecanoyl-glycero-3-phosphoserine	metab_10076	-	576.27	5.04	M+K-2H	C25H50NO9P	1.45	1.27	0.00
529	neg_3873	PE(22:6(4Z,7Z,10Z,13Z,16Z,19Z)/16:0)	metab_10660	-	762.51	6.35	M-H	C43H74NO8P	1.05	1.27	0.01
530	pos_5507	Linalool oxide D 3-[apiosyl-(1->6)-glucoside]	metab_4894	-	506.26	3.47	M+ACN+H	C21H36O11	1.40	1.27	0.00
531	neg_4979	Estrone	metab_15418	481-97-0;53-16-7	585.33	5.52	2M+FA-H	C18H22O2	1.46	1.27	0.01
532	pos_3453	Physagulin F	metab_2672	-	509.26	5.53	M+H-2H2O	C30H40O9	1.23	1.27	0.01
533	neg_4994	Physangulide	metab_11861	131749-58-1	567.28	5.49	M+FA-H	C28H42O9	1.52	1.27	0.00

534	pos_5989	3-Amino-4-methylpentanoic acid	metab_5428	5699-54-7	326.21	2.67	2M+ACN+Na	C6H13NO2	1.29	1.28	0.01
535	neg_5433	Ibritumomab tiuxetan	metab_12343	-	590.21	4.37	M+Na-2H	C24H35N5O11	1.37	1.28	0.01
536	pos_2616	Endomorphin-1	metab_1746	189388-22-5	628.33	2.88	M+NH4	C34H38N6O5	1.38	1.28	0.00
537	pos_815	Adenosine, 8-(butylamino)-N-cyclopentyl-	metab_7443	-	407.24	5.06	M+H, M+Na	C19H30N6O4	1.30	1.28	0.01
538	neg_636	Asp Leu Ala Glu	metab_13327	-	445.19	2.38	M-H, M+Na-2H	C18H30N4O9	1.46	1.28	0.03
539	pos_2850	Penitrem A	metab_2004	12627-35-9	651.32	3.39	M+NH4	C37H44ClNO6	1.43	1.28	0.00
540	pos_5202	Trp Glu Val	metab_4560	-	433.21	4.12	M+H	C21H28N4O6	1.27	1.28	0.00
541	neg_5458	DIPROTIN A	metab_12368	-	340.22	4.33	M-H	C17H31N3O4	1.32	1.28	0.01
542	neg_5719	Mibefradil	metab_12653	116644-53-2	532.24	3.89	M+K-2H	C29H38FN3O3	1.44	1.28	0.01
543	pos_7434	5-Oxohexanoylcarnitine	metab_6965	-	337.17	0.62	M+ACN+Na	C13H23NO5	1.23	1.28	0.03
544	neg_2662	DIPROTIN B	metab_9362	-	326.21	3.60	M-H	C16H29N3O4	1.36	1.28	0.02
545	pos_3264	Lidamidine	metab_2462	-	463.25	4.72	2M+Na	C11H16N4O	1.35	1.28	0.00
546	pos_2855	2-(2-Aminopropanylamino)-4-carbamoylbutyric acid	metab_2009	-	435.22	3.40	2M+H	C8H15N3O4	1.33	1.28	0.00
547	pos_6583	Val Glu Ser	metab_6067	-	334.16	1.94	M+H	C13H23N3O7	1.34	1.28	0.01
548	pos_6190	Pectenotoxin 1	metab_5650	97564-90-4	438.24	2.40	M+2H	C47H70O15	1.45	1.28	0.00
549	pos_3132	Pyrimethanil	metab_2317	53112-28-0	462.23	4.21	2M+ACN+Na	C12H13N3	1.24	1.28	0.01
550	neg_5407	(E)-indol-3-ylacetaldoxime	metab_12315	-	521.23	4.41	3M-H	C10H10N2O	1.40	1.28	0.02

551	neg_6517	Ile Ser Thr Glu	metab_13492	-	447.21	2.77	M-H	C18H32N4O9	1.52	1.28	0.01
552	neg_6567	3-Fluorophenmetrazine	metab_13545	-	389.20	2.70	2M-H	C11H14FNO	1.42	1.28	0.04
553	pos_2067	Ser Leu Ser Gly Leu	metab_1153	-	476.27	1.84	M+H	C20H37N5O8	1.38	1.28	0.00
554	pos_1223	Val Gly Thr	metab_249	-	276.15	1.83	M+H-H2O, M+H	C11H21N3O5	1.38	1.28	0.01
555	neg_5927	Pentanoic acid, 5-(dipentylamino)-5-oxo-4-((3-quinolinylcarbonyl)amino)-, (R)-	metab_12879	-	486.26	3.55	M+FA-H	C25H35N3O4	1.45	1.28	0.01
556	pos_573	N-Acetylproline	metab_5141	68-95-1	332.18	2.56	M+ACN+Na, 2M+NH4	C7H11NO3	1.33	1.28	0.02
557	neg_5507	Janthitrem F	metab_12422	90986-52-0	690.37	4.25	M+FA-H	C39H51NO7	1.68	1.28	0.00
558	pos_2849	Adouetine Y	metab_2002	19542-38-2	632.32	3.39	M+ACN+Na	C34H40N4O4	1.40	1.29	0.00
559	pos_3005	Ile Pro Ile	metab_2176	-	342.24	3.82	M+H	C17H31N3O4	1.33	1.29	0.00
560	pos_5917	Persicaxanthin	metab_5349	80952-82-5	429.23	2.79	M+2Na-H	C25H36O3	1.39	1.29	0.01
561	pos_5523	Protocrocin	metab_4912	-	809.36	3.43	M+2Na	C76H116O34	1.47	1.29	0.00
562	pos_4228	2-Deoxycastasterone	metab_3500	87734-68-7	431.35	6.49	M+H-H2O	C28H48O4	1.36	1.29	0.00
563	neg_4881	Cortisol 21-sulfate	metab_11737	1253-43-6	883.33	5.63	2M-H	C21H30O8S	1.59	1.29	0.00
564	neg_5068	17-Dimethylaminogeldanamycin	metab_11943	-	615.34	5.24	M-H	C32H48N4O8	1.49	1.29	0.00

565	neg_6989	Glycylhydroxyproline	metab_13974	24587-32-4	375.15	2.08	2M-H	C7H12N2O4	1.43	1.29	0.00
566	pos_4918	Phe Met Leu	metab_4246	-	410.21	4.87	M+H	C20H31N3O4S	1.27	1.29	0.00
567	neg_380	Leu Ile Ser Glu	metab_10584	-	459.25	4.56	M-H, M+FA-H	C20H36N4O8	1.41	1.29	0.02
568	pos_2750	3-Amino-2,3-dihydrobenzoic acid	metab_1894	87980-11-8	320.16	3.17	2M+ACN+H	C7H9NO2	1.38	1.29	0.00
569	neg_5439	Sulfolithocholylglycine	metab_15435	474-74-8;15324-64-8	512.27	4.35	M-H	C26H43NO7S	1.47	1.29	0.00
570	neg_6231	(2Z)-2-Morpholin-4-yliminoacetonitrile	metab_13191	-	416.21	3.14	3M-H	C6H9N3O	1.44	1.29	0.01
571	pos_6419	Threonyllysine	metab_5892	23161-31-1	517.30	2.09	2M+Na	C10H21N3O4	1.39	1.29	0.01
572	neg_5803	Pindolol	metab_12743	13523-86-9	541.30	3.77	2M+FA-H	C14H20N2O2	1.46	1.29	0.00
573	neg_2751	Dihydroxylysinonorleucine	metab_9458	-	472.24	3.78	M+Na-2H	C18H37N5O8	1.43	1.29	0.04
574	pos_2640	Asp Gly Lys Ile Leu	metab_1773	-	545.33	2.94	M+H	C24H44N6O8	1.37	1.30	0.01
575	pos_6422	Leu Glu Thr	metab_5896	-	362.19	2.09	M+H	C15H27N3O7	1.37	1.30	0.00
576	pos_2364	Valyl-prolyl-glycyl-valyl-glycine	metab_1471	-	428.25	2.39	M+H	C19H33N5O6	1.33	1.30	0.02
577	pos_2382	Pro Glu Ile	metab_1491	-	358.20	2.42	M+H	C16H27N3O6	1.32	1.30	0.01
578	pos_6982	Trospectomycin	metab_6488	-	419.18	1.17	M+2Na-H	C17H30N2O7	1.38	1.30	0.00
579	neg_5740	N-Jasmonoylisoleucine	metab_12677	120330-93-0	344.18	3.86	M+Na-2H	C18H29NO4	1.46	1.30	0.03
580	pos_1885	N1,N12-Diacetylspermine	metab_959	61345-83-3	331.21	1.27	M+2Na-H	C14H30N4O2	1.37	1.30	0.01
581	neg_3003	LysoPE(0:0/20:5(5Z,8Z,11Z,14Z,17Z))	metab_9732	-	498.26	4.27	M-H	C25H42NO7P	1.40	1.30	0.02

582	neg_3369	N-Arachidonoyl Alanine	metab_10130	-	356.26	5.21	M-H2O-H	C23H37NO3	1.53	1.30	0.00
583	pos_632	Val Ala Val	metab_5789	-	288.19	3.24	M+H, M+Na	C13H25N3O4	1.44	1.30	0.00
584	pos_2717	Val Asn Ile	metab_1857	-	345.21	3.11	M+H	C15H28N4O5	1.43	1.30	0.00
585	pos_2305	Gly Phe Asn	metab_1409	-	337.15	2.27	M+H	C15H20N4O5	1.40	1.30	0.01
586	pos_5718	Ile Arg Leu	metab_5128	-	401.29	3.12	M+H	C18H36N6O4	1.35	1.30	0.00
587	pos_5923	Tert-Butyl (2-(2-hydroxyethoxy)ethyl)carbamate	metab_5356	-	449.22	2.77	2M+K	C9H19NO4	1.47	1.30	0.00
588	neg_5006	Vertilmicin	metab_11875	-	470.30	5.46	M-H2O-H	C22H43N5O7	1.49	1.30	0.02
589	neg_6434	Lysylhydroxyproline	metab_13408	870233-57-1	304.15	2.88	M+FA-H	C11H21N3O4	1.44	1.30	0.00
590	neg_5838	Fluphenazine enanthate	metab_12781	2746-81-8	530.25	3.71	M-H2O-H	C29H38F3N3O2S	1.44	1.30	0.01
591	pos_3076	2-(p-Acetamidophenyl)-2-ethylglutarimide	metab_2254	-	316.17	4.02	M+ACN+H	C15H18N2O3	1.36	1.30	0.00
592	pos_2829	Beta-Cortol	metab_1981	667-65-2	401.29	3.35	M+CH3OH+H	C21H36O5	1.41	1.30	0.00
593	pos_728	Ile Glu Ile	metab_6798	-	374.23	4.04	M+H-2H2O, M+H	C17H31N3O6	1.34	1.30	0.01
594	pos_6218	3-O-Demethylfortimicin A	metab_5681	-	414.23	2.36	M+Na	C16H33N5O6	1.40	1.30	0.00
595	neg_4991	(9Z,11E,14Z)-(13S)-hydroperoxyoctadeca-(9,11,14)-trienoate	metab_11858	-	354.20	5.50	M+FA-H	C18H29O4-	1.45	1.30	0.00
596	pos_2684	Asiaticoside	metab_1820	16830-15-2	502.25	3.04	M+2Na	C48H78O19	1.36	1.30	0.01
597	pos_5425	Arginine, ethyl ester	metab_4805	-	443.25	3.63	2M+K	C8H18N4O2	1.43	1.30	0.00

598	neg_5684	Lysyl-aspartyl-glutamyl-leucine	metab_12614	-	502.25	3.95	M-H	C21H37N5O9	1.46	1.30	0.05
599	neg_2840	Vorapaxar	metab_9555	-	473.23	3.95	M-H2O-H	C29H33FN2O4	1.41	1.30	0.04
600	neg_5552	Leustroducsin B	metab_12472	-	704.33	4.16	M+Cl	C34H56NO10P	1.54	1.30	0.00
601	neg_2156	D-Fucitol	metab_8828	-	331.16	2.77	2M-H	C6H14O5	1.53	1.31	0.01
602	pos_6174	Ala Val Val	metab_5632	-	288.19	2.43	M+H	C13H25N3O4	1.39	1.31	0.00
603	pos_6007	Prostaglandin D1	metab_5450	17968-82-0	387.27	2.64	M+CH3OH+H	C20H34O5	1.38	1.31	0.00
604	neg_3419	Suloctidil	metab_10185	-	372.21	5.38	M+Cl	C20H35NOS	1.47	1.31	0.01
605	pos_5821	PE(14:1(9Z)/20:5(7Z,9Z,11E,13E,17Z)-3OH(5,6,15))	metab_5243	-	794.40	2.96	M+K	C39H66NO11P	1.47	1.31	0.00
606	pos_2771	Thr Pro Phe	metab_1917	-	364.19	3.23	M+H	C18H25N3O5	1.41	1.31	0.00
607	neg_2900	Allyl-isopropyl-acetylharnstoff	metab_9621	-	427.26	4.07	2M+Hac-H	C9H16N2O2	1.39	1.31	0.04
608	neg_5537	S-(11-hydroxy-9-deoxy-delta12-PGD2)-glutathione	metab_12455	-	644.33	4.20	M-H	C30H51N3O10S	1.34	1.31	0.04
609	pos_6229	Ser Asp Ile	metab_5693	-	334.16	2.35	M+H	C13H23N3O7	1.42	1.31	0.01
610	neg_3498	Ancistrocladine	metab_10269	32221-59-3	406.20	5.58	M-H	C25H29NO4	1.50	1.31	0.01
611	pos_6084	Thr Gln Leu	metab_5533	-	361.21	2.55	M+H	C15H28N4O6	1.39	1.31	0.01
612	pos_6387	Ribostamycin	metab_5859	25546-65-0	419.21	2.13	M+H-2H2O	C17H34N4O10	1.52	1.31	0.00
613	neg_5534	Psychosine sulfate	metab_12452	-	586.28	4.20	M+FA-H	C24H47NO10S	1.48	1.31	0.05
614	pos_2354	Val Ser Val	metab_1460	-	304.19	2.37	M+H	C13H25N3O5	1.38	1.31	0.00
615	pos_6349	Pro Leu Lys	metab_5821	-	357.25	2.18	M+H	C17H32N4O4	1.49	1.31	0.00

616	pos_6344	Lys Val Leu	metab_5816	-	359.26	2.19	M+H	C17H34N4O4	1.41	1.31	0.00
617	pos_5673	Ile Lys Leu	metab_5078	-	373.28	3.19	M+H	C18H36N4O4	1.45	1.31	0.00
618	neg_5451	Linalool 3,6-oxide primeveroside	metab_12361	-	463.22	4.34	M-H	C21H36O11	1.39	1.31	0.02
619	pos_5827	Ile Gly Val	metab_5249	-	288.19	2.95	M+H	C13H25N3O4	1.42	1.32	0.00
620	pos_2678	25-Hydroxyvitamin D2 25-(beta-glucuronide)	metab_1813	-	627.33	3.03	M+K	C34H52O8	1.47	1.32	0.00
621	neg_6812	Val Glu Asn Glu	metab_13799	-	488.20	2.38	M-H	C19H31N5O10	1.56	1.32	0.00
622	pos_2950	Preladenant	metab_2115	-	521.27	3.66	M+NH4	C25H29N9O3	1.43	1.32	0.00
623	pos_7435	Thr Gly Arg	metab_6966	-	333.19	0.62	M+H	C12H24N6O5	1.36	1.32	0.01
624	neg_6471	Aminodeoxykanamycin	metab_13443	2251180.00	504.23	2.83	M+Na-2H	C18H37N5O10	1.57	1.32	0.01
625	neg_1586	Serylmethionine	metab_8257	484927.00	235.08	1.68	M-H	C8H16N2O4S	1.48	1.32	0.04
626	pos_509	Prolyl-Gamma-glutamate	metab_4435	-	276.16	2.44	M+CH3OH+H, 2M+NH4	C10H17N3O4	1.48	1.32	0.00
627	neg_3417	N-(3-Methylbut-2-EN-1-YL)-9H-purin-6-amine	metab_10183	-	405.23	5.38	2M-H	C10H13N5	1.46	1.32	0.01
628	pos_5674	L-Isoleucine	metab_5079	1509-34-8;73-32-5	173.13	3.19	M+ACN+H	C6H13NO2	1.31	1.32	0.00
629	neg_3222	Semagacestat	metab_9969	-	406.20	4.78	M+FA-H	C19H27N3O4	1.45	1.32	0.02
630	neg_2282	P-Chlorodisopyramide	metab_8965	-	372.19	2.96	M-H	C21H28ClN3O	1.51	1.32	0.02
631	pos_6039	CDP-DG(PGD2/i-22:0)	metab_5484	-	578.80	2.60	M+H+Na	C54H93N3O18P2	1.51	1.32	0.00
632	pos_6037	Val Lys Leu	metab_5482	-	359.26	2.61	M+H	C17H34N4O4	1.45	1.32	0.00
633	neg_6044	Beraprost	metab_13008	-	433.18	3.37	M+Cl	C24H30O5	1.40	1.32	0.03

634	pos_5719	Gly Leu Val	metab_5129	-	288.19	3.12	M+H	C13H25N3O4	1.48	1.32	0.00
635	neg_6547	Bursopoietin	metab_13524	-	360.18	2.73	M+Na-2H	C14H25N7O3	1.47	1.32	0.04
636	neg_5688	Rotundifoline	metab_12618	-	399.19	3.94	M-H	C22H28N2O5	1.41	1.32	0.02
637	pos_708	Ile Gly Leu	metab_6588	-	302.21	3.84	M+H, M+Na	C14H27N3O4	1.43	1.33	0.00
638	pos_5386	Ile Glu Leu Lys	metab_4761	-	502.32	3.72	M+H	C23H43N5O7	1.47	1.33	0.00
639	pos_6767	Dioxolane guanosine	metab_6258	-	524.20	1.71	2M+NH4	C9H11N5O4	1.49	1.33	0.00
640	pos_5534	Cyclic 3-Hydroxymelatonin	metab_4924	-	281.15	3.43	M+CH3OH+H	C13H16N2O3	1.34	1.33	0.00
641	pos_2498	Tramadol	metab_1616	27203-92-5	286.18	2.63	M+Na	C16H25NO2	1.49	1.33	0.00
642	neg_6534	(9S,10S)-10-hydroxy-9-(phosphonooxy)octadecanoate	metab_13511	-	417.20	2.75	M+Na-2H	C18H37O7P	1.67	1.33	0.00
643	pos_5722	Val Glu Leu Asp	metab_5133	-	475.24	3.11	M+H	C20H34N4O9	1.44	1.33	0.00
644	pos_6744	Laninamivir	metab_6234	203120-17-6	388.18	1.76	M+ACN+H	C13H22N4O7	1.44	1.33	0.01
645	neg_5750	17-Aminogeldanamycin	metab_12688	-	544.26	3.85	M-H	C28H39N3O8	1.55	1.33	0.02
646	neg_2918	Trilostane	metab_9640	13647-35-3	350.17	4.11	M+Na-2H	C20H27NO3	1.56	1.33	0.01
647	neg_7214	2-Methyl-2-oxazoline	metab_14207	-	169.10	1.76	2M-H	C4H7NO	1.47	1.33	0.01
648	pos_3046	Oxadixyl	metab_2221	77732-09-3	320.16	3.93	M+ACN+H	C14H18N2O4	1.41	1.33	0.00
649	neg_625	Blasticidin S	metab_13208	2079-00-7	403.18	2.11	M-H2O-H, M+Na-2H	C17H26N8O5	1.61	1.33	0.01
650	pos_2533	Ile Ile Lys	metab_1656	-	373.28	2.71	M+H	C18H36N4O4	1.44	1.33	0.00
651	pos_5958	N(6)-L-Homocysteinyl-L-lysine	metab_5394	-	544.30	2.72	2M+NH4	C10H21N3O3S	1.45	1.33	0.01

652	neg_3450	N-Acetyl-9-aminominocycline, (4R)-	metab_10219	-	432.18	5.51	M+Cl	C20H31NO7	1.62	1.33	0.00
653	pos_3053	Asn Ala Glu Val Leu	metab_2229	-	545.29	3.95	M+H	C23H40N6O9	1.47	1.34	0.00
654	pos_1835	Gln Glu Ala	metab_905	-	347.16	1.14	M+H	C13H22N4O7	1.50	1.34	0.00
655	pos_6688	5'-Deoxy-5-fluorocytidine	metab_6178	-	278.12	1.86	M+CH3OH+H	C9H12FN3O4	1.35	1.34	0.02
656	neg_5452	1-Piperidinyloxy, 4-(((dodecyloxy)hydroxyphosphinyl)oxy)-2,2,6,6-tetramethyl-	metab_12362	-	442.27	4.34	M+Na-2H	C21H44NO5P	1.42	1.34	0.04
657	pos_6751	Cis-3-Hexenyl b-primeveroside	metab_6242	132278-37-6	412.22	1.73	M+NH4	C17H30O10	1.51	1.34	0.00
658	neg_3395	METHIONYL-LEUCYLPHENYLALANINE	metab_10159	-	408.20	5.29	M-H	C20H31N3O4S	1.50	1.34	0.01
659	pos_4805	Neurotensin(1-11)	metab_4123	-	723.88	5.34	M+2H	C66H99N19O18	1.49	1.34	0.00
660	pos_5107	Glu Phe Ile	metab_4455	-	408.21	4.34	M+H	C20H29N3O6	1.38	1.34	0.01
661	pos_2804	Heliotridine	metab_1954	520-63-8	328.22	3.31	2M+NH4	C8H13NO2	1.38	1.34	0.00
662	pos_5632	PA(10:0/PGF2alpha)	metab_5033	-	701.35	3.25	M+K	C33H59O11P	1.49	1.34	0.01
663	pos_5964	L-Prolinamide, glycyl-L-prolyl-L-arginyl-	metab_5401	-	389.24	2.71	M+H-2H2O	C18H32N8O4	1.47	1.34	0.00
664	pos_2752	(Z)-2-Methyl-2-butene-1,4-diol 4-O-beta-D-Glucopyranoside	metab_1896	-	546.28	3.17	2M+NH4	C11H20O7	1.52	1.34	0.00
665	neg_2618	Z-FA-Fmk	metab_9314	-	771.32	3.52	2M-H	C21H23FN2O4	1.51	1.34	0.02
666	neg_379	Cholylthreonine	metab_10572	-	544.30	4.58	M+Cl, M+K-2H	C28H47NO7	1.55	1.34	0.02

667	neg_6644	Eriojaposide B	metab_13626	351412-97-0	561.25	2.58	M+FA-H	C25H40O11	1.63	1.34	0.01
668	neg_2736	Homoharringtonine	metab_9442	-	580.23	3.76	M+Cl	C29H39NO9	1.50	1.34	0.02
669	neg_888	6-O-Desmethyldonepezil	metab_15281	-	364.19	3.77	M-H2O-H, M-H	C23H27NO3	1.53	1.34	0.00
670	pos_5181	Ethinamate	metab_4537	126-52-3	376.22	4.16	2M+ACN+H	C9H13NO2	1.49	1.34	0.00
671	pos_5177	MURABUTIDE	metab_4532	-	590.30	4.17	M+ACN+H	C23H40N4O11	1.43	1.34	0.00
672	pos_2846	Mycophenolate mofetil	metab_7668	24280-93-1;128794-94-5	475.24	3.39	M+ACN+H	C23H31NO7	1.47	1.34	0.00
673	pos_733	Leu Ser Ile	metab_6852	-	332.22	4.09	M+H, M+Na	C15H29N3O5	1.45	1.34	0.01
674	pos_5870	7-Oxociguatoxin	metab_5297	263336-55-6	564.29	2.88	M+2H	C60H86O20	1.47	1.34	0.00
675	neg_5445	PA(8:0/22:6(5Z,7Z,10Z,13Z,16Z,19Z)-OH(4))	metab_12354	-	661.29	4.34	M+K-2H	C33H53O9P	1.47	1.35	0.00
676	pos_5998	Butenoylcarnitine	metab_5438	-	293.15	2.66	M+ACN+Na	C11H19NO4	1.44	1.35	0.00
677	neg_7301	8beta-Angeloyloxy-15-hydroxy-1alpha,10R-dimethoxy-3-oxo-11(13)-germacren-12,6alpha-olide	metab_14282	-	445.18	1.53	M+Na-2H	C22H32O8	1.65	1.35	0.00
678	neg_2040	Diacetoxyscirpenol	metab_8713	2270-40-8	365.16	2.56	M-H	C19H26O7	1.47	1.35	0.03
679	neg_855	Valencic acid	metab_15245	-	617.28	3.56	2M+FA-H, 3M-H	C12H14O3	1.57	1.35	0.01
680	pos_2383	Gln Val Tyr	metab_1492	-	409.21	2.42	M+H	C19H28N4O6	1.48	1.35	0.00
681	pos_5651	Cortol	metab_5054	516-38-1	401.29	3.23	M+CH3OH+H	C21H36O5	1.47	1.35	0.00
682	neg_1801	Valylasparagine	metab_8464	66170-00-1	230.11	2.08	M-H	C9H17N3O4	1.58	1.35	0.01

683	neg_7409	D-ononitol	metab_14388	6090-97-7;24333-07-1	433.16	1.22	2M+FA-H	C7H14O6	1.67	1.35	0.00
684	pos_5972	Thr Glu Ile	metab_5410	-	362.19	2.70	M+H	C15H27N3O7	1.55	1.35	0.00
685	neg_7218	Asparaginyl-Methionine	metab_14209	-	262.09	1.74	M-H	C9H17N3O4S	1.56	1.35	0.01
686	pos_5695	DG(10:0/20:5(5Z,8Z,11Z,14Z,16E)-OH(18R)/0:0)	metab_5102	-	585.36	3.15	M+K	C33H54O6	1.62	1.35	0.00
687	pos_5975	Ethyl 7-epi-12-hydroxyjasmonate glucoside	metab_5413	-	381.19	2.69	M+H-2H2O	C20H32O9	1.41	1.35	0.01
688	neg_2496	PA(PGD1/10:0)	metab_9182	-	697.35	3.27	M+Cl	C33H59O11P	1.53	1.35	0.02
689	neg_6313	Nonylurea	metab_13277	-	185.17	3.03	M-H	C10H22N2O	1.38	1.35	0.02
690	pos_5412	CDP-DG(22:6(4Z,8Z,10Z,13Z,16Z,19Z)-OH(7)/i-22:0)	metab_4791	-	563.81	3.66	M+2H	C56H93N3O16P2	1.44	1.35	0.01
691	pos_5555	Apramycin	metab_4947	37321-09-8	504.27	3.39	M+H-2H2O	C21H41N5O11	1.54	1.35	0.01
692	neg_4964	1-(13Z,16Z-docosadienoyl)-glycero-3-phosphate	metab_11829	-	471.28	5.54	M-H2O-H	C25H47O7P	1.59	1.36	0.01
693	neg_396	1-[Fluoro(methoxy)phosphoryl]oxyicosa-5,8,11,14-tetraene	metab_10748	-	421.21	4.91	M+Cl, M+K-2H	C21H36FO3P	1.52	1.36	0.02
694	pos_5988	Labetalol	metab_5427	36894-69-6	351.17	2.67	M+Na	C19H24N2O3	1.43	1.36	0.01
695	pos_5511	Arg Leu Phe	metab_4899	-	435.27	3.46	M+H	C21H34N6O4	1.43	1.36	0.01
696	neg_3012	DG(20:4(6E,8Z,11Z,13E)-2OH(5S,15S)/2:0/0:0)	metab_9742	-	497.27	4.29	M+FA-H	C25H40O7	1.53	1.36	0.02

697	neg_5408	Ala Gly Leu Val Ser	metab_12316	-	444.25	4.41	M-H	C19H35N5O7	1.55	1.36	0.02
698	pos_4763	4(1H)-Pyridinone, 2-ethyl-3-hydroxy-1-(2-hydroxyethyl)-	metab_4077	-	430.19	5.47	2M+ACN+Na	C9H13NO3	1.38	1.36	0.00
699	pos_6655	Gamma-Glutamyl-beta-aminopropiononitrile	metab_6145	-	232.13	1.89	M+CH3OH+H	C8H13N3O3	1.48	1.36	0.00
700	pos_2049	Gly Leu Arg	metab_1135	-	345.22	1.78	M+H	C14H28N6O4	1.55	1.36	0.00
701	pos_3207	Leu-leu-ome	metab_2399	-	259.20	4.45	M+H	C13H26N2O3	1.47	1.36	0.00
702	neg_2767	2-Benzyl-2-hydroxybutanamide	metab_9474	-	445.23	3.82	2M+Hac-H	C11H15NO2	1.51	1.36	0.03
703	pos_2263	Leu Glu Lys Glu	metab_1364	-	518.28	2.18	M+H	C22H39N5O9	1.49	1.36	0.00
704	pos_571	Docebenone	metab_5119	80809-81-0	716.39	2.82	M+CH3OH+H, 2M+ACN+Na	C21H26O3	1.49	1.36	0.01
705	pos_2252	Kanamycin	metab_7663	59-01-8;8063-07-8	449.22	2.14	M+H-2H2O	C18H36N4O11	1.47	1.36	0.01
706	pos_2946	Gln Ile Ile	metab_2110	-	373.24	3.65	M+H	C17H32N4O5	1.47	1.36	0.00
707	pos_4158	SM(d18:0/16:1(9Z))	metab_3427	-	703.57	7.00	M+H	C39H79N2O6P	1.28	1.36	0.00
708	pos_3199	Isoforskolin	metab_2389	-	393.22	4.43	M+H-H2O	C22H34O7	1.37	1.36	0.02
709	neg_5904	2-dehydropantoate	metab_12854	-	437.17	3.59	3M-H	C6H10O4	1.51	1.37	0.02
710	neg_5194	1,2-Dihexanoyl-sn-glycero-3-phosphoethanolamine	metab_12082	-	392.18	4.88	M-H2O-H	C17H34NO8P	1.42	1.37	0.03
711	pos_5392	Leu Lys Phe	metab_4768	-	407.26	3.71	M+H	C21H34N4O4	1.43	1.37	0.00
712	pos_5948	Gln Leu Thr Asp	metab_5383	-	476.23	2.74	M+H	C19H33N5O9	1.48	1.37	0.01

713	neg_3325	Antiarrhythmic peptide	metab_10083	-	451.20	5.06	M-H2O-H	C19H30N6O8	1.67	1.37	0.00
714	pos_562	Ala Thr Leu Asp	metab_5019	-	419.21	2.67	M+H, M+ACN+H	C17H30N4O8	1.59	1.37	0.00
715	neg_5308	Nelfinavir	metab_12206	159989-64-7	588.29	4.62	M+Na-2H	C32H45N3O4S	1.57	1.37	0.01
716	neg_5263	Tryptophyl-Glycine	metab_12157	-	521.22	4.72	2M-H	C13H15N3O3	1.53	1.37	0.01
717	pos_5617	Val Asp Leu	metab_5016	-	346.20	3.29	M+H	C15H27N3O6	1.57	1.37	0.00
718	neg_3011	10,11-dihydro-20-trihydroxy-leukotriene B4	metab_9741	-	431.23	4.29	M+FA-H	C20H34O7	1.51	1.37	0.03
719	pos_5453	S-(PGA2)-glutathione	metab_4836	-	659.33	3.57	M+NH4	C30H47N3O10S	1.53	1.37	0.01
720	neg_3131	MG(LTE4/0:0/0:0)	metab_9870	-	558.28	4.53	M+FA-H	C26H43NO7S	1.58	1.37	0.02
721	pos_2503	PI(TXB2/16:0)	metab_1623	-	474.26	2.63	M+H+Na	C45H81O17P	1.60	1.37	0.00
722	pos_5443	Antibiotic A 204A	metab_4825	-	495.27	3.59	M+2Na	C49H84O17	1.54	1.37	0.00
723	neg_2895	7-Sulfocholic acid	metab_9614	60320-05-0	487.24	4.06	M-H	C24H40O8S	1.58	1.37	0.03
724	neg_5175	Oleuropein	metab_12062	32619-42-4	539.18	4.92	M-H	C25H32O13	1.80	1.37	0.00
725	neg_6970	(2R,3R,4R)-Hexane-1,2,3,4,6-pentol	metab_13956	-	331.16	2.10	2M-H	C6H14O5	1.67	1.37	0.01
726	pos_3371	8-methylthiooctyldesulfoglucosinolate	metab_2581	-	430.19	5.28	M+CH3OH+H	C16H31NO6S2	1.46	1.37	0.00
727	pos_1896	Astromicin	metab_971	55779-06-1	388.25	1.31	M+H-H2O	C17H35N5O6	1.50	1.37	0.00
728	neg_5857	Floratheasaponin E	metab_12802	-	642.31	3.67	M-2H	C63H98O27	1.54	1.37	0.02

729	neg_742	Glycylproline	metab_14398	704-15-4	515.25	2.91	2M+FA-H, 3M-H	C7H12N2O3	1.59	1.38	0.01
730	pos_3224	(2S)-2-(2-Benzamidoethylamino)-5-(diaminomethylideneamino)pentanoic acid	metab_2418	-	660.39	4.50	2M+NH4	C15H23N5O3	1.58	1.38	0.00
731	pos_2716	Chakasaponin V	metab_1856	-	644.32	3.11	M+2H	C63H98O27	1.58	1.38	0.00
732	neg_3275	PC(2:0/PGF1alpha)	metab_10027	-	672.32	4.91	M+Cl	C30H56NO11P	1.60	1.38	0.01
733	pos_2809	PS(22:4(7Z,10Z,13Z,16Z)/22:5(7Z,10Z,13Z,16Z,19Z))	metab_1959	-	465.77	3.31	M+2Na	C50H80NO10P	1.51	1.38	0.01
734	neg_1491	Kuguacin B	metab_8164	-	417.16	1.35	M-H	C25H23FN2O3	1.66	1.38	0.01
735	pos_3187	Phe Ser Ile	metab_2376	-	366.20	4.37	M+H	C18H27N3O5	1.42	1.38	0.00
736	pos_4954	Ile Ile Leu	metab_4286	-	358.27	4.76	M+H	C18H35N3O4	1.51	1.38	0.00
737	neg_6050	Isofloxythepin	metab_13015	-	445.19	3.36	M+FA-H	C23H29FN2OS	1.60	1.39	0.02
738	pos_2128	Asn Ile Gln	metab_1219	-	374.20	1.94	M+H	C15H27N5O6	1.53	1.39	0.00
739	neg_7441	Oxiracetam	metab_14420	-	375.15	1.14	2M+Hac-H	C6H10N2O3	1.62	1.39	0.01
740	neg_5954	4-Aminohex-5-ynoic acid	metab_12909	-	380.18	3.52	3M-H	C6H9NO2	1.54	1.39	0.01
741	neg_6963	2-Amino-3-methyl-1-pyrrolidin-1-YL-butan-1-one	metab_13948	-	215.14	2.10	M+FA-H	C9H18N2O	1.56	1.39	0.01
742	neg_5349	Implitapide	metab_12251	-	530.28	4.53	M-H	C35H37N3O2	1.56	1.39	0.04

743	neg_5456	Actein	metab_12366	-	697.35	4.33	M+Na-2H	C37H56O11	1.54	1.39	0.01
744	neg_6586	Gln Val Asp Glu	metab_13564	-	488.20	2.68	M-H	C19H31N5O10	1.70	1.39	0.00
745	pos_5455	APGPR Enterostatin	metab_4837	117830-79-2	461.26	3.57	M+H-2H2O	C21H36N8O6	1.51	1.39	0.00
746	pos_5855	Lys Ile Leu	metab_5280	-	373.28	2.91	M+H	C18H36N4O4	1.50	1.39	0.01
747	pos_4953	Calpeptin	metab_4285	117591-20-5	380.25	4.76	M+NH4	C20H30N2O4	1.37	1.40	0.02
748	pos_4817	Glu Phe Leu	metab_4135	-	408.21	5.28	M+H	C20H29N3O6	1.45	1.40	0.00
749	pos_1125	Val Ile Ile	metab_140	-	344.25	4.31	M+H, M+Na	C17H33N3O4	1.54	1.40	0.00
750	pos_6759	Gly Lys Ile	metab_6250	-	317.22	1.72	M+H	C14H28N4O4	1.59	1.40	0.00
751	pos_5274	DG(6 keto-PGF1alpha/0:0/2:0)	metab_4638	-	504.32	3.96	M+NH4	C25H42O9	1.61	1.40	0.00
752	neg_6455	Linatine	metab_13430	10139-06-7	517.23	2.84	2M-H	C10H17N3O5	1.65	1.40	0.00
753	neg_7057	Amabiline	metab_14047	17958-43-9	304.15	2.03	M+Na-2H	C15H25NO4	1.70	1.40	0.00
754	pos_2766	[(2S,3R)-2-Amino-3-hydroxybutanoyl] (2S)-pyrrolidine-2-carboxylate	metab_1911	-	474.26	3.21	2M+ACN+H	C9H16N2O4	1.59	1.40	0.01
755	pos_3143	Prolyl-Valine	metab_2329	-	451.25	4.24	2M+Na	C10H18N2O3	1.47	1.40	0.01
756	pos_2641	1-Methyladenosine	metab_1774	15763-06-1	580.26	2.94	2M+NH4	C11H15N5O4	1.58	1.40	0.00
757	neg_2588	Desacetoxyvindoline	metab_9282	-	841.44	3.46	2M+FA-H	C23H30N2O4	1.66	1.40	0.01
758	pos_6819	Cinncassiol C3	metab_6309	64979-94-8	383.20	1.61	M+H	C20H30O7	1.53	1.40	0.00
759	neg_4921	(3R)-Acetoxy-(5S)-hydroxy-1-(4-hydroxy-3-methoxyphenyl)-decane	metab_11782	-	359.18	5.59	M+Na-2H	C19H30O5	1.54	1.40	0.02
760	pos_2587	Cilligen	metab_1714	-	504.23	2.81	M+ACN+H	C22H30N4O5S	1.55	1.41	0.01

761	pos_4829	Cepharanthine	metab_4148	481-49-2	620.28	5.20	2M+3H2O+2H	C37H38N2O6	1.59	1.41	0.00
762	pos_99	Buprenorphine 3-beta-D-Glucuronide	metab_7635	-	685.38	4.37	M+ACN+H, M+ACN+Na, 2M+3H2O+2H	C35H49NO10	1.66	1.41	0.00
763	neg_3228	Cucurbitacin D	metab_9975	3877-86-9	553.26	4.80	M+K-2H	C30H44O7	1.67	1.41	0.00
764	neg_5387	PGP(18:2(10E,12Z)+=O(9)/16:1(9Z))	metab_12293	-	883.44	4.44	M+FA-H	C40H72O14P2	1.65	1.41	0.01
765	pos_5282	Ile Asp Ile	metab_4647	-	360.21	3.95	M+H	C16H29N3O6	1.55	1.41	0.00
766	neg_2432	Farnesylcysteine	metab_9120	68000-92-0	360.18	3.18	M+Cl	C18H31NO2S	1.73	1.41	0.01
767	pos_6732	Ala Ile Arg	metab_6222	-	359.24	1.81	M+H	C15H30N6O4	1.63	1.41	0.00
768	neg_5299	S-(PGA1)-glutathione	metab_12195	-	688.32	4.63	M+FA-H	C30H49N3O10S	1.62	1.41	0.02
769	pos_350	4-[(Hydroxymethyl)nitrosoamino]-1-(3-pyridinyl)-1-butanone	metab_2724	-	447.20	1.51	M+ACN+Na, 2M+H	C10H13N3O3	1.64	1.41	0.00
770	pos_5625	Capecitabine	metab_5025	154361-50-9	377.18	3.27	M+NH4	C15H22FN3O6	1.55	1.42	0.01
771	pos_6821	Ectoine	metab_6312	96702-03-3	326.18	1.61	2M+ACN+H	C6H10N2O2	1.59	1.42	0.00
772	pos_5121	Leu Glu Ile	metab_4471	-	374.23	4.31	M+H	C17H31N3O6	1.54	1.42	0.00
773	pos_5292	Phe Asp Ile Glu	metab_4658	-	523.24	3.92	M+H	C24H34N4O9	1.55	1.42	0.01
774	neg_2537	Glaucarubin	metab_9226	1448-23-3	541.23	3.35	M+FA-H	C25H36O10	1.68	1.42	0.00
775	pos_5145	4-Hydroxypropranolol glucuronide	metab_4497	-	920.40	4.25	2M+NH4	C22H29NO9	1.47	1.42	0.04
776	pos_5228	Leu Asp Leu	metab_4587	-	360.21	4.07	M+H	C16H29N3O6	1.56	1.42	0.00
777	neg_2431	5-Hydroxylysinonorleucine	metab_9119	-	312.16	3.18	M+Na-2H	C12H25N3O5	1.61	1.42	0.01

778	pos_5327	Hydroxyprolyl-Valine	metab_4697	-	502.29	3.84	2M+ACN+H	C10H18N2O4	1.55	1.42	0.01
779	pos_5465	9,13-Dihydroxy-4-megastigmen-3-one 9-glucoside	metab_4848	188305-07-9	421.24	3.55	M+CH3OH+H	C19H32O8	1.39	1.42	0.03
780	neg_5357	Kinetensin 4-7	metab_12260	138482-56-1	592.26	4.50	M+Na-2H	C26H37N9O6	1.73	1.42	0.00
781	pos_6008	Val Glu Val	metab_5451	-	346.20	2.64	M+H	C15H27N3O6	1.60	1.42	0.01
782	neg_2719	Garcinol	metab_9423	78824-30-3	583.35	3.72	M-H2O-H	C38H50O6	1.76	1.42	0.00
783	pos_2705	Gentamicin C2	metab_1844	25876-11-3	486.29	3.09	M+Na	C20H41N5O7	1.62	1.43	0.00
784	pos_781	Asn Phe Trp	metab_7376	-	466.21	4.60	M+H, M+Na	C24H27N5O5	1.51	1.43	0.00
785	neg_5374	10,11-Dihydro-12R-hydroxy-leukotriene E4	metab_12279	-	502.25	4.48	M+FA-H	C23H39NO6S	1.69	1.43	0.02
786	pos_5926	Thr Pro Ile	metab_5359	-	330.20	2.77	M+H	C15H27N3O5	1.46	1.43	0.04
787	pos_5397	Val Val Ile	metab_4773	-	330.24	3.70	M+H	C16H31N3O4	1.53	1.43	0.00
788	pos_2586	Pyridinoline	metab_1713	63800-01-1	461.22	2.81	M+CH3OH+H	C18H28N4O8	1.61	1.43	0.01
789	pos_5817	N-Eicosapentaenoyl Proline	metab_5238	-	444.24	2.97	M+2Na-H	C25H37NO3	1.58	1.43	0.01
790	pos_3059	Ile Gly Ile	metab_2235	-	302.21	3.97	M+H	C14H27N3O4	1.60	1.43	0.01
791	pos_3084	Dimerum acid	metab_2263	-	502.29	4.05	M+NH4	C22H36N4O8	1.56	1.43	0.01
792	pos_786	Val Phe Ile	metab_7410	-	378.24	4.63	M+H, M+Na	C20H31N3O4	1.54	1.43	0.00
793	pos_3284	Val Phe Phe	metab_2484	-	412.22	4.79	M+H	C23H29N3O4	1.51	1.43	0.00
794	neg_3198	5-O-Desmethyldonepezil	metab_9941	-	364.19	4.71	M-H	C23H27NO3	1.62	1.43	0.01
795	pos_846	Gly Leu Phe	metab_7477	-	336.19	4.52	M+H, M+Na	C17H25N3O4	1.52	1.44	0.01
796	pos_5858	Rigin	metab_5283	77727-17-4	520.26	2.90	M+ACN+Na	C18H32N8O6	1.64	1.44	0.00
797	pos_5636	17a-Ethynylestradiol	metab_5037	57-63-6	360.19	3.25	M+ACN+Na	C20H24O2	1.60	1.44	0.00
798	pos_7022	Glycylprolylarginine	metab_6528	-	329.19	1.12	M+H	C13H24N6O4	1.64	1.44	0.00

799	neg_5395	Bisphenol B	metab_12302	77-40-7	543.28	4.43	2M+Hac-H	C16H18O2	1.68	1.44	0.01
800	neg_1926	4-[(2R,5R)-5-(6-Aminopurin-9-yl)-3,4-dihydroxyoxolan-2-yl]butanamide	metab_8590	-	703.29	2.36	2M+Hac-H	C13H18N6O4	1.75	1.45	0.00
801	pos_2646	(1(10)E,4a,5E)-1(10),5-Germacradiene-12-acetoxy-4,11-diol	metab_1779	143305-08-2	615.38	2.96	2M+Na	C17H28O4	1.65	1.45	0.00
802	pos_5456	3-Isobutyl-1-methylxanthine	metab_4838	-	508.24	3.56	2M+ACN+Na	C10H14N4O2	1.56	1.45	0.01
803	neg_3058	3-Benzyl-5-(6-carboxyhexyl)-1-(2-cyclohexyl-2-hydroxyethylamino)hydantoin	metab_9788	-	440.25	4.37	M-H2O-H	C25H37N3O5	1.68	1.45	0.02
804	pos_7115	S-Triazine, 2-amino-4-((4-methyl-1-piperazinyl)methyl)-6-piperidino-	metab_6627	-	274.21	0.98	M+H-H2O	C14H25N7	1.63	1.45	0.01
805	pos_2801	Dynorphin A (6-8)	metab_1951	-	488.27	3.30	M+2Na-H	C18H37N9O4	1.62	1.45	0.01
806	pos_749	Ala Ile Phe	metab_7025	-	350.21	4.25	M+H, M+Na	C18H27N3O4	1.52	1.45	0.01
807	pos_5479	Prolylproline	metab_4863	20488-28-2	442.27	3.52	2M+NH4	C10H16N2O3	1.60	1.45	0.00
808	pos_3114	Leu Glu Leu	metab_2297	-	374.23	4.16	M+H	C17H31N3O6	1.58	1.45	0.01
809	neg_5687	1-heptadecanoyl-glycero-3-phosphate	metab_12617	-	461.21	3.94	M+K-2H	C20H41O7P	1.70	1.45	0.01
810	neg_1733	Dihydrozeatin-O-glucoside	metab_8394	62512-96-3	382.17	2.00	M-H	C16H25N5O6	1.71	1.45	0.00

811	pos_3438	Ganglioside GD3 (d18:1/9Z-18:1)	metab_2655	-	757.91	5.50	M+2Na	C70H123N3O29	1.64	1.45	0.00
812	neg_4966	Goshonoside F6	metab_11831	138614-60-5	615.34	5.54	M-H	C31H52O12	1.74	1.45	0.00
813	neg_5424	(2Z)-2-(3-Phenylpropoxyimino)butanoic acid	metab_12334	-	469.23	4.38	2M-H	C13H17NO3	1.68	1.45	0.00
814	neg_5289	Hordatine B	metab_12184	10502-21-3	617.26	4.66	M+K-2H	C29H40N8O5	1.68	1.46	0.01
815	pos_2698	Ile Lys Ile	metab_1835	-	373.28	3.08	M+H	C18H36N4O4	1.57	1.46	0.00
816	pos_3209	Vaniprevir	metab_2401	-	780.35	4.46	M+Na	C38H55N5O9S	1.57	1.46	0.03
817	neg_6512	((4-(4-Amidinophenoxy)butanoyl)aspartyl)valine	metab_13487	-	481.19	2.77	M+FA-H	C20H28N4O7	1.70	1.46	0.01
818	pos_5734	Threoninyl-Valine	metab_5146	99032-17-4	500.27	3.10	2M+ACN+Na	C9H18N2O4	1.64	1.46	0.00
819	pos_5621	Etiracetam	metab_5021	102767-28-2	382.24	3.28	2M+ACN+H	C8H14N2O2	1.56	1.46	0.01
820	pos_5195	20-Hydroxy-leukotriene E4	metab_4551	111844-33-8	488.27	4.13	M+CH3OH+H	C23H37NO6S	1.58	1.46	0.01
821	neg_5416	Chymostatin	metab_12325	9076-44-2	588.29	4.39	M-H2O-H	C31H41N7O6	1.66	1.46	0.01
822	neg_2864	Alpha-Fluoromethylhistamine	metab_9581	-	428.25	3.99	3M-H	C6H10FN3	1.68	1.46	0.01
823	pos_6233	Prenyl glucoside	metab_5698	117861-55-9	560.27	2.34	2M+ACN+Na	C11H20O6	1.67	1.47	0.00
824	neg_5042	Janthitrem G	metab_11915	90986-51-9	610.36	5.33	M-H2O-H	C39H51NO6	1.82	1.47	0.00
825	pos_5380	Ser Leu Leu	metab_4755	-	332.22	3.74	M+H	C15H29N3O5	1.45	1.47	0.04

826	neg_5053	Aconitinum	metab_11927	-	644.31	5.28	M-H	C34H47NO11	1.78	1.47	0.00
827	pos_6622	Ile Gly Lys	metab_6109	-	317.22	1.92	M+H	C14H28N4O4	1.64	1.47	0.01
828	neg_619	DG(12:0/20:5(7Z,9Z,11E,13E,17Z)-3OH(5,6,15)/0:0)	metab_13155	-	643.37	5.55	M+Na-2H, M+K-2H	C35H58O8	1.75	1.47	0.01
829	neg_5612	Nalbuphine	metab_12538	20594-83-6	378.17	4.07	M+Na-2H	C21H27NO4	1.60	1.48	0.01
830	neg_5176	Piperolein B	metab_12063	30505-89-6	364.19	4.92	M+Na-2H	C21H29NO3	1.77	1.48	0.02
831	pos_2753	Thr Val Leu	metab_1897	-	332.22	3.18	M+H	C15H29N3O5	1.67	1.48	0.00
832	neg_5127	PS(5-iso PGF2VI/14:0)	metab_12009	-	812.41	5.05	M+Cl	C38H68NO13P	1.96	1.48	0.00
833	pos_5449	Gly Ile Phe	metab_4831	-	336.19	3.59	M+H	C17H25N3O4	1.55	1.48	0.00
834	pos_774	Thr Val Phe	metab_7299	-	366.20	4.48	M+H, M+Na	C18H27N3O5	1.62	1.48	0.01
835	neg_2849	3-Aminopropyl(diethoxymethyl)phosphinic acid	metab_9564	-	674.34	3.97	3M-H	C8H20NO4P	1.62	1.48	0.03
836	pos_2903	PA(LTE4/18:4(6Z,9Z,12Z,15Z))	metab_2063	-	437.72	3.53	M+H+Na	C44H70NO11PS	1.58	1.48	0.01
837	pos_6088	Gentamicin A sulfate	metab_5537	-	433.23	2.54	M+H-2H2O	C18H36N4O10	1.63	1.48	0.02
838	pos_2304	Murametide	metab_1408	-	548.26	2.26	M+ACN+H	C20H34N4O11	1.72	1.48	0.00
839	pos_5882	Versetamide	metab_5310	-	490.25	2.87	M+H-H2O	C20H37N5O10	1.71	1.48	0.00
840	pos_5458	Ser Val Phe	metab_4840	-	352.19	3.56	M+H	C17H25N3O5	1.64	1.49	0.00
841	pos_100	Ala Leu Phe	metab_2	-	350.21	4.51	M+H, 2M+ACN+H, M+Na	C18H27N3O4	1.67	1.49	0.00
842	pos_6225	Butirosina	metab_5689	-	520.26	2.35	M+H-2H2O	C21H41N5O12	1.72	1.49	0.00

843	pos_5793	Ile Asp Val Glu	metab_5211	-	475.24	3.00	M+H	C20H34N4O9	1.69	1.49	0.01
844	neg_6217	2-Hydroxy-desipramine glucuronide	metab_13181	-	479.18	3.14	M+Na-2H	C24H30N2O7	1.82	1.49	0.00
845	pos_2971	Plazomicin	metab_2138	-	656.36	3.71	M+ACN+Na	C25H48N6O10	1.69	1.49	0.00
846	pos_2347	PIP(20:2(11Z,14Z)/TXB2)	metab_1452	-	529.26	2.35	M+2H	C49H86O20P2	1.71	1.49	0.00
847	pos_5207	Ile Gly Phe	metab_4565	-	336.19	4.11	M+H	C17H25N3O4	1.55	1.49	0.01
848	pos_2367	8-Hydroxymianserin	metab_1474	-	602.35	2.39	2M+ACN+H	C18H20N2O	1.67	1.49	0.00
849	pos_5287	Ser Leu Ile	metab_4652	-	332.22	3.93	M+H	C15H29N3O5	1.64	1.50	0.00
850	neg_3281	24,24-Dfhv	metab_10034	-	471.28	4.93	M+Cl	C27H42F2O2	1.70	1.50	0.02
851	neg_5760	Ile-Val-Val	metab_12699	-	328.22	3.83	M-H	C16H31N3O4	1.71	1.50	0.01
852	pos_4945	Capsicoside E1	metab_4276	109575-83-9	607.29	4.79	M+2H	C56H92O28	1.71	1.50	0.00
853	pos_5074	PI(22:6(4Z,7Z,10Z,12E,16Z,19Z)-OH(14)/22:3(10Z,13Z,16Z))	metab_4418	-	518.28	4.41	M+2Na	C54H87O14P	1.62	1.50	0.00
854	neg_2642	N-Lauroyl Tryptophan	metab_9340	-	385.25	3.57	M-H	C23H34N2O3	1.72	1.50	0.01
855	neg_701	(-)-2-Difluoromethylornithine	metab_13998	-	545.26	2.69	2M+FA-H, 3M-H	C6H12F2N2O2	1.84	1.50	0.01
856	pos_6202	Strictosidine	metab_5664	20824-29-7	548.26	2.38	M+NH4	C27H34N2O9	1.76	1.50	0.00
857	pos_5275	Leu Gly Phe	metab_4639	-	336.19	3.96	M+H	C17H25N3O4	1.58	1.50	0.01
858	pos_5728	Alanylvaline	metab_5139	3303-45-5	440.25	3.11	2M+ACN+Na	C8H16N2O3	1.61	1.50	0.01
859	neg_5198	Virginiamycin m1	metab_12086	21411-53-0	506.23	4.87	M-H2O-H	C28H35N3O7	1.80	1.50	0.01
860	pos_6057	Beta-Alanyl-L-arginine	metab_5503	-	529.26	2.58	2M+K	C9H19N5O3	1.73	1.50	0.00
861	neg_2682	Coutaric acid	metab_9383	-	394.20	3.64	M+FA-H	C18H27N3O4	1.66	1.50	0.01
862	pos_2091	Ala Val Thr	metab_1180	-	290.17	1.87	M+H	C12H23N3O5	1.65	1.51	0.01

863	pos_775	Ile Val Ile	metab_7310	-	344.25	4.49	M+H, M+Na	C17H33N3O4	1.65	1.51	0.01
864	pos_3043	Gln Leu Leu	metab_2218	-	373.24	3.92	M+H	C17H32N4O5	1.66	1.51	0.00
865	pos_2611	Val Val Val	metab_1741	-	316.22	2.87	M+H	C15H29N3O4	1.67	1.51	0.00
866	neg_5380	Linalool 3,7-oxide beta-primeveroside	metab_12286	-	463.22	4.46	M-H	C21H36O11	1.76	1.51	0.01
867	neg_2925	Valylproline	metab_9647	20488-27-1	473.26	4.12	2M+FA-H	C10H18N2O3	1.73	1.51	0.02
868	neg_5637	Colforsin daropate	metab_12564	-	546.24	4.02	M+K-2H	C27H43NO8	1.75	1.52	0.00
869	pos_2882	Glycinamide, glycyl-L-prolyl-	metab_2039	-	474.28	3.49	2M+NH4	C9H16N4O3	1.72	1.52	0.00
870	pos_827	8-Pentanoylneosolaniol	metab_7456	116163-74-7	489.21	5.28	M+H, M+Na	C24H34O9	1.66	1.52	0.00
871	pos_2964	Caffeine	metab_2130	21399.00	430.20	3.70	2M+ACN+H	C8H10N4O2	1.66	1.53	0.00
872	neg_3154	Dynorphin B (6-9)	metab_9895	-	626.32	4.59	M+Na-2H	C26H43N11O6	1.86	1.53	0.00
873	pos_2333	Gamma-L-Glutamyl-L-pipecolic acid	metab_1439	-	276.15	2.33	M+NH4	C11H18N2O5	1.66	1.53	0.00
874	neg_3479	Aplaviroc	metab_10248	-	576.30	5.56	M-H	C33H43N3O6	1.83	1.54	0.00
875	pos_3423	Nafarelin	metab_2639	76932-56-4	661.83	5.49	M+2H	C66H83N17O13	1.74	1.54	0.00
876	neg_3503	Fosteabine	metab_10276	73532-83-9	574.33	5.58	M-H	C27H50N3O8P	1.82	1.54	0.00
877	neg_5655	Capsianside A	metab_12584	116107-40-5	781.39	3.99	M-2H	C76H124O33	1.97	1.54	0.00
878	neg_5438	Hordatine A	metab_12348	7073-64-5	587.27	4.35	M+Na-2H	C28H38N8O5	1.81	1.55	0.01
879	pos_6276	11-beta-Hydroxyandrosterone-3-glucuronide	metab_7646	-	515.28	2.28	M+CH3OH+H	C25H38O9	1.70	1.55	0.00

880	pos_5599	Leu Lys Leu	metab_4995	-	373.28	3.31	M+H	C18H36N4O4	1.74	1.55	0.01
881	pos_3082	Val Ile Leu	metab_2261	-	344.25	4.04	M+H	C17H33N3O4	1.70	1.55	0.00
882	neg_3153	D-Glucamine	metab_9894	-	542.28	4.59	3M-H	C6H15NO5	1.86	1.55	0.01
883	pos_5387	Leu Gly Leu	metab_4762	-	302.21	3.72	M+H	C14H27N3O4	1.69	1.55	0.00
884	pos_2355	Thr Val Val	metab_1461	-	318.20	2.37	M+H	C14H27N3O5	1.62	1.55	0.03
885	neg_3300	LysoPE(22:2(13Z,16Z)/0:0)	metab_10056	-	554.32	4.98	M+Na-2H	C27H52NO7P	1.87	1.55	0.00
886	pos_5676	Teleocidin B 2	metab_5081	-	490.29	3.18	M+K	C28H41N3O2	1.76	1.55	0.00
887	neg_2671	Alkergot	metab_9371	-	628.30	3.62	M+K-2H	C33H45N5O5	1.84	1.57	0.00
888	pos_5508	Val Glu Leu	metab_4895	-	360.21	3.47	M+H	C16H29N3O6	1.70	1.57	0.02
889	neg_1414	Guanosine diphosphate	metab_8089	146-91-8	442.02	1.10	M-H	C10H15N5O11P2	1.78	1.57	0.03
890	pos_2873	Hoduloside X	metab_2029	154971-14-9	558.28	3.44	M+H+Na	C53H88O23	1.85	1.59	0.00
891	pos_3302	Araloside S1	metab_2505	256531-73-4	623.32	4.87	M+2Na	C61H100O23	1.82	1.60	0.00
892	pos_5375	Arjunolic acid 3-glucoside	metab_4750	99543-11-0	673.39	3.74	M+Na	C36H58O10	1.88	1.60	0.00
893	pos_2570	Telocinobufagin	metab_1696	472-26-4	447.21	2.77	M+2Na-H	C24H34O5	1.76	1.61	0.01
894	neg_2322	AFN911	metab_9006	-	546.24	3.03	M+Cl	C29H33N7O2	1.91	1.62	0.03
895	pos_5248	Nummularine A	metab_4609	53947-95-8	680.41	4.01	M+CH3OH+H	C36H49N5O6	1.97	1.63	0.00
896	pos_5424	Deoxycholic acid 3-glucuronide	metab_4804	72504-58-6	551.32	3.63	M+H-H2O	C30H48O10	1.76	1.63	0.00
897	pos_4811	PI(22:6(5Z,8E,10Z,13Z,15E,19Z)-2OH(7S,17S)/20:4(8Z,11Z,14Z,17Z))	metab_4129	-	927.50	5.31	M+H-2H2O	C51H79O15P	1.86	1.63	0.00
898	pos_791	Ala Leu Leu	metab_7416	-	338.20	4.71	M+H, M+Na	C15H29N3O4	1.81	1.63	0.00
899	pos_5758	Ser Val Leu	metab_5172	-	318.20	3.07	M+H	C14H27N3O5	1.70	1.64	0.01
900	pos_4815	Arachidonic acid	metab_4133	73836-78-9	514.29	5.64	M+NH4	C25H40N2O6S	1.36	1.64	0.00

901	pos_2786	Antigonadotropic decapeptide	metab_1933	-	616.78	3.26	M+2Na	C58H81N11O16	1.93	1.66	0.00
902	pos_5460	Milbemycin alpha9	metab_4843	-	701.33	3.55	M+ACN+Na	C36H47NO9	1.91	1.66	0.00
903	pos_5233	Ile Gln Leu	metab_4593	-	373.24	4.04	M+H	C17H32N4O5	1.83	1.67	0.01
904	neg_5231	Yakuchinone-A	metab_12124	78954-23-1	683.36	4.80	2M+Hac-H	C20H24O3	2.05	1.67	0.00
905	pos_3106	1-Adamantanecarbonyl-RF-NH2	metab_2288	-	546.31	4.12	M+ACN+Na	C26H38N6O3	1.87	1.68	0.00
906	pos_1069	Phytosphingosine	metab_78	554-62-1	318.30	5.89	M+H-H2O, M+H	C18H39NO3	1.98	1.68	0.00
907	pos_3040	Isoleucyl-Threonine	metab_2215	-	528.30	3.91	2M+ACN+Na	C10H20N2O4	1.85	1.70	0.01
908	pos_5454	Coclaurine	metab_7655	486-39-5	593.26	3.57	2M+Na	C17H19NO3	1.89	1.71	0.00
909	pos_5784	PA(10:0/PGE1)	metab_5201	-	701.35	3.01	M+K	C33H59O11P	1.82	1.71	0.01
910	pos_744	Ile Thr Ile	metab_6971	-	346.23	4.18	M+H, M+Na	C16H31N3O5	1.81	1.71	0.01
911	pos_5078	H-Tyr-Phe-Leu-Phe-Arg-Pro-Arg-Asn-NH2	metab_4422	-	556.31	4.40	M+2H	C54H78N16O10	1.93	1.75	0.00
912	neg_3213	Lyciumoside III	metab_9959	-	629.35	4.76	M-H2O-H	C32H56O13	1.87	1.75	0.04
913	neg_7014	Thr Val Ala Lys	metab_14003	-	416.25	2.07	M-H	C18H35N5O6	2.15	1.76	0.00
914	pos_826	Hebevinoside III	metab_7455	89203-39-4	817.48	5.28	M+H, M+Na	C43H70O13	2.02	1.79	0.00
915	pos_5683	Ile Gln Val	metab_5089	-	359.23	3.17	M+H	C16H30N4O5	1.90	1.79	0.01
916	neg_4850	Taurin	metab_11703	-	285.09	5.68	M+K-2H	C15H20O3	1.92	1.80	0.00
917	neg_5361	Petromyzonol sulfate	metab_12265	-	473.26	4.50	M-H	C24H42O7S	1.95	1.81	0.01
918	pos_3226	CDP-DG(22:3(10Z,13Z,16Z)/20:5(	metab_2420	-	554.79	4.51	M+2H	C55H87N3O16P2	2.02	1.86	0.00

		5Z,8Z,11Z,14Z,16E)-OH(18))									
919	pos_5982	Leu Ala Ser Ser Thr	metab_5421	-	478.25	2.68	M+H	C19H35N5O9	2.03	1.86	0.00
920	neg_2926	Deoxynivalenol	metab_9648	51481-10-8	591.25	4.12	2M-H	C15H20O6	2.15	1.86	0.00
921	pos_3025	Ile Ala Ile	metab_2198	-	316.22	3.87	M+H	C15H29N3O4	1.90	1.88	0.01
922	neg_2996	Heliotron	metab_9722	-	671.37	4.25	2M+FA-H	C16H27NO5	2.16	1.90	0.00
923	pos_3287	Cyclolinopeptide B	metab_2487	-	540.79	4.80	M+H+Na	C56H83N9O9S	2.17	1.90	0.00
924	pos_4934	Asparagoside A	metab_4264	14835-43-9	617.35	4.82	M+K	C33H54O8	1.92	1.90	0.00
925	neg_3111	1-Oleoylglycerophosphoserine	metab_9848	-	544.26	4.48	M+Na-2H	C24H46NO9P	2.04	1.92	0.02
926	neg_4975	Indinavir	metab_11841	150378-17-9	658.36	5.53	M+FA-H	C36H47N5O4	2.14	1.94	0.00
927	pos_5790	Ile Ala Val	metab_5208	-	302.21	3.01	M+H	C14H27N3O4	1.76	1.97	0.02
928	neg_3466	Clarithromycin	metab_10236	81103-11-9	768.45	5.53	M+Na-2H	C38H69NO13	2.14	1.98	0.01
929	neg_5285	Glycyl-Histidine	metab_12180	-	635.27	4.67	3M-H	C8H12N4O3	2.00	2.03	0.03
930	pos_2808	Met Val Glu	metab_1958	-	378.17	3.31	M+H	C15H27N3O6S	1.81	2.04	0.04
931	neg_2567	PS(14:0/14:1(9Z))	metab_9259	-	714.38	3.40	M+K-2H	C34H64NO10P	2.09	2.09	0.02
932	pos_796	Schidigerasaponin B1	metab_7421	266997-32-4	885.46	4.79	M+H, M+Na	C44H68O18	2.25	2.11	0.00
933	pos_7605	PE(18:4(6Z,9Z,12Z,15Z)/24:1(15Z))	metab_7153	-	274.87	0.54	M+3H	C47H84NO8P	1.85	2.15	0.02
934	pos_5047	Mucronine D	metab_4388	38496-00-3	694.42	4.47	M+CH3OH+H	C37H51N5O6	2.26	2.30	0.00
935	pos_5416	Desglucocheirotoxol	metab_4795	29336-13-8	570.32	3.65	M+NH4	C29H44O10	2.27	2.66	0.00

936	neg_391	Pentacarboxylporphyrin I	metab_10698	28100-78-9	697.25	4.82	M-H, M+Na-2H	C37H38N4O10	2.80	2.80	0.00
937	pos_5172	Pirbuterol	metab_4527	38677-81-5	522.33	4.19	2M+ACN+H	C12H20N2O3	2.32	3.12	0.00
938	neg_3132	Ergocristine	metab_9871	511-08-0	630.26	4.53	M+Na-2H	C35H39N5O5	2.61	3.23	0.00
939	pos_4901	Leucylproline	metab_4228	6403-35-6	520.31	4.92	2M+ACN+Na	C11H20N2O3	2.50	4.27	0.00
940	pos_5277	Milataxel	metab_4641	-	854.37	3.95	M+H	C44H55NO16	2.90	8.66	0.00
